# A novel bioinformatics strategy to uncover the active ingredients and molecular mechanisms of Bai Shao in the treatment of non-alcoholic fatty liver disease

**DOI:** 10.3389/fphar.2024.1406188

**Published:** 2024-06-28

**Authors:** Shuaibing He, Hantao Chen, Yanfeng Yi, Diandong Hou, Xuyan Fu, Jinlu Xie, Juan Zhang, Chongbin Liu, Xiaochen Ru, Juan Wang

**Affiliations:** ^1^ Key Laboratory of Vector Biology and Pathogen Control of Zhejiang Province, School of Medicine, Huzhou Central Hospital, Huzhou University, Huzhou, China; ^2^ Key Laboratory for Precise Prevention and Control of Major Chronic Diseases, Huzhou University, Huzhou, China; ^3^ Department of Life Sciences and Health, School of Science and Engineering, Huzhou College, Huzhou, China; ^4^ XinJiang Institute of Chinese Materia Medica and Ethnodrug, Urumqi, China; ^5^ School of Traditional Chinese Medicine, Zhejiang Pharmaceutical University, Ningbo, China

**Keywords:** bioinformatics, Bai Shao, non-alcoholic fatty liver disease, Traditional Chinese Medicine, hepatoprotection, network pharmacology, active ingredient, molecular mechanism

## Abstract

**Introduction:** As a new discipline, network pharmacology has been widely used to disclose the material basis and mechanism of Traditional Chinese Medicine in recent years. However, numerous researches indicated that the material basis of TCMs identified based on network pharmacology was the mixtures of beneficial and harmful substances rather than the real material basis. In this work, taking the anti-NAFLD (non-alcoholic fatty liver disease) effect of Bai Shao (BS) as a case, we attempted to propose a novel bioinformatics strategy to uncover the material basis and mechanism of TCMs in a precise manner.

**Methods:** In our previous studies, we have done a lot work to explore TCM-induced hepatoprotection. Here, by integrating our previous studies, we developed a novel computational pharmacology method to identify hepatoprotective ingredients from TCMs. Then the developed method was used to discover the material basis and mechanism of Bai Shao against Non-alcoholic fatty liver disease by combining with the techniques of molecular network, microarray data analysis, molecular docking, and molecular dynamics simulation. Finally, literature verification method was utilized to validate the findings.

**Results:** A total of 12 ingredients were found to be associated with the anti-NAFLD effect of BS, including monoterpene glucosides, flavonoids, triterpenes, and phenolic acids. Further analysis found that IL1-β, IL6, and JUN would be the key targets. Interestingly, molecular docking and molecular dynamics simulation analysis showed that there indeed existed strong and stable binding affinity between the active ingredients and the key targets. In addition, a total of 23 NAFLD-related KEGG pathways were enriched. The major biological processes involved by these pathways including inflammation, apoptosis, lipid metabolism, and glucose metabolism. Of note, there was a great deal of evidence available in the literature to support the findings mentioned above, indicating that our method was reliable.

**Discussion:** In summary, the contributions of this work can be summarized as two aspects as follows. Firstly, we systematically elucidated the material basis and mechanism of BS against NAFLD from multiple perspectives. These findings further enhanced the theoretical foundation of BS on NAFLD. Secondly, a novel computational pharmacology research strategy was proposed, which would assist network pharmacology to uncover the scientific connotation TCMs in a more precise manner.

## 1 Introduction

Non-alcoholic fatty liver disease (NAFLD) is defined as unusual hepatic fat accumulation in the absence of excessive alcohol consumption and any other conditions that may lead to hepatic steatosis (Chalasani et al., 2012). Patients suffered from NAFLD always presented liver dysfunction and are confronted with higher risk of liver-related morbidity (Mantovani et al., 2020). It was reported that NAFLD has been a leading cause of cirrhosis, hepatocellular carcinoma, and some other irreversible liver diseases (Powell et al., 2021). In addition, NAFLD also increased the risk of cardiovascular diseases (Yoshitaka et al., 2017), chronic kidney diseases (Mantovani et al., 2022), and malignancy (Rinella and Sanyal, 2016) significantly. In fact, NAFLD is a newly discovered disease. It has not been described until 1980s. However, in just a couple of decades, it has become a global health issue because of its high prevalence (Siegel et al., 2012; Perumpail et al., 2017). Epidemiological research showed that the morbidity of NAFLD in many countries was already close to 30% (Younossi et al., 2016; Estes et al., 2018). Although the liver disease specialists have made a tremendous amount of effort to seek effective therapeutic drugs for NAFLD during the past decades, there is no approved therapy until now. Therefore, scientific researches focused on exploring the candidate drugs of NAFLD deserve to be paid a great deal of attention.

Paeoniae Radix Alba, the dried roots of *Paeonia lactiflora* Pall., is a well-known herbal medicine and widely used in Asia for a long history. Owning to it presented very obvious effect on some complex diseases, it has been recorded in the Pharmacopoeias of many countries, including but not limit to China and Japan. In China, Paeoniae Radix Alba is known as Bai Shao (BS) and mainly used to regulate menstruation, antiperspirant, relieve pain and protect liver (Chinese Pharmacopeia Commission, 2020). In recent years, with the social and economic burdens caused by NAFLD increasing continually, the hepatoprotective effect of BS gradually gained more and more attention. It has been demonstrated that BS and its extracts have multiple beneficial effects on liver, including drug/chemical-induced liver injury attenuation, anti-fatty liver, anti-hepatitis, anti-hepatic fibrosis, cholestasis alleviation, and hepatocellular carcinoma inhibition (Ma et al., 2020). Especially for the action of preventing and treating NAFLD, a great deal of evidence has been provided in previous publications. Sun et al. established fatty liver model by intervening HepG2 cells with oleic acid and explored the anti-fatty liver action of BS. As a result, they found that compared with the model group, the levels of TC, TG, ALP, and ALT of the pretreatment group and the treatment group were decreased significantly (Sun et al., 2022). The anti-fatty liver effect of BS in animal models has also been confirmed (Zhang et al., 2015; Ma et al., 2016). For example, Ma et al. found that BS treatment can obviously ameliorate the histopathological and biochemical changes in NAFLD rats induced by high-fat diet via inhibiting lipid ectopic deposition (Ma et al., 2017). In addition, some clinical scholars have attempted to treat NAFLD by combining polyene phosphatidylcholine and the total glycosides of BS. Excitedly, the total effective rates of the combination group increased by 47.06% in comparison with the polyene phosphatidylcholine treatment group. Besides, more significant improvements on clinical symptoms, liver function, and blood lipids were also observed in the combination group (Tian et al., 2014). In summary, the anti-NAFLD effect of BS has been well documented. However, which compounds are the active ingredients and how they produce these beneficial effects have not been fully elucidated. Therefore, more research focused on revealing the mechanisms of BS against NAFLD is needed.

Different from the western medicine of “one target, one drug,” as complex mixtures of many bioactive ingredients, Traditional Chinese Medicines (TCMs) always produce their effects via multi-component and multi-target, making it to be a challenging work to unveil the material basis and mechanism of TCM systemically (Li, 2016). In 2007, network pharmacology, a new discipline, was proposed by Hopkins AL (Hopkins, 2008). Unlike the reductionism research strategies which treat both drugs and targets in isolation, network pharmacology attempts to illustrate the interactions between drugs and biological systems in a systematic manner (Kibble et al., 2015). The perspective of network pharmacology is in accord with the holistic theory of TCM, making it possible to deeply understand the scientific connotation of TCM. Furthermore, compared with the conventional methods, network pharmacology has the advantages of high-efficacy, effort-saving, and low cost. During the past decade, network pharmacology has attracted increasing attention and has become a popular tool in the field of TCM research (Li and Zhang, 2013). However, in the current state of the art, there still existed some problems in the field of network pharmacology. For example, in a research from Henan University of Traditional Chinese Medicine, the authors attempted to illustrate the material basis of Psoraleae Fructus-induced liver injury. Network pharmacology analysis indicated that a total of 25 compounds were associated with the hepatotoxicity of Psoraleae Fructus (Cao et al., 2022). However, we found that not all of these substances were harmful to the liver. Inversely, quite a few of them were liver-friendly and considered to be promising hepatoprotectors, such as daidzein, biochanin A, and delphinidin. Numerous data showed that these compounds have the effects of anti-NAFLD (Kim et al., 2011; Fan et al., 2021), anti-hepatic fibrosis (Domitrović and Jakovac, 2010; Breikaa et al., 2013b), anti-hepatocellular carcinoma (Xiao et al., 2020; Zakaria et al., 2021; Sun et al., 2023) and attenuating liver injury induced by multiple chemical agents or drugs (Domitrović and Jakovac, 2010; Breikaa et al., 2013a; Yu et al., 2020). In addition, both *in-vivo* and *in silico* toxicity studies indicated that daidzein is a safe natural substance without hepatotoxicity (Laddha et al., 2022). In another network pharmacology study from the University of Hong Kong, kaempferol and thymol exhibited the largest number of liver injury targets connections and considered to be play a crucial role in Xiao Chai Hu Tang-induced liver injury (Hong et al., 2017). However, both kaempferol (Ren et al., 2019; Xiao et al., 2022; Akiyama et al., 2023) and thymol (Jafari et al., 2018; Guo et al., 2023; Lahmi et al., 2023) are promising and safe agents for treating multiple liver diseases. They have never been implicated in the adverse events of drug-induced liver injury. In fact, the similar phenomena were broadly existed in network pharmacology researches (Wang et al., 2017; Zheng et al., 2020; Li et al., 2021). Cases mentioned above suggested that not all of the compounds connected with liver injury targets were hepatotoxic substances. Inversely, they could be liver-friendly substances. Likewise, we speculated that the compounds connected with liver disease targets were the mixtures of hepatotoxic and hepatoprotective substances. Therefore, in the network pharmacology researches focused on revealing the material basis and mechanism of TCM-induced hepatoprotection, establishing a method to identify the hepatoprotective ingredients may help to improve the precision of the results.

**FIGURE 1:**
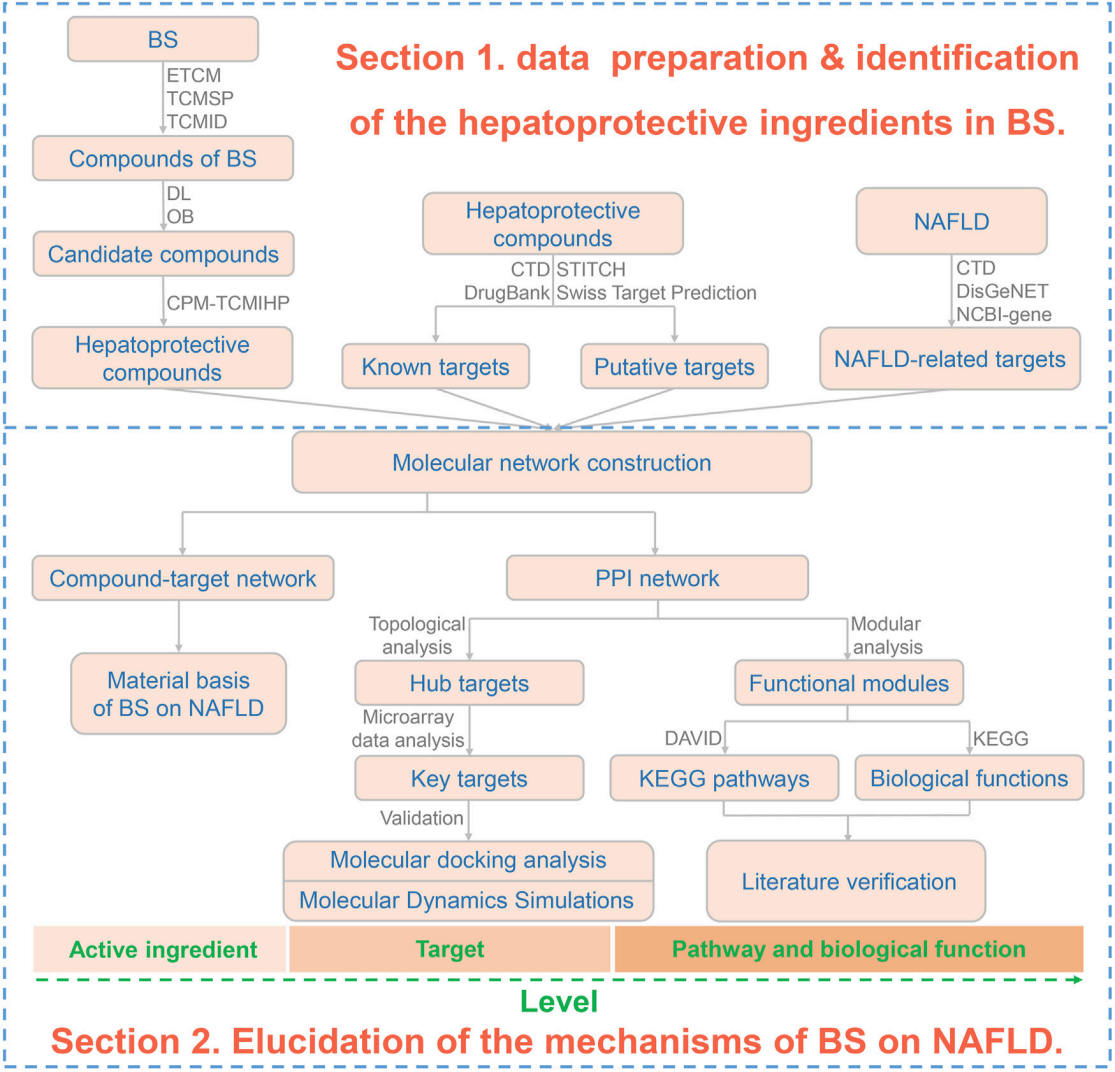
Flowchart of the overall methodology. BS, Bai Shao; DL, drug likeness; OB, oral bioavailability; NAFLD, non-alcoholic fatty liver disease; PPI, protein-protein interaction. CPM-TCMIHP indicated the computational pharmacology method for identifying the material basis of TCM-induced hepatoprotection which were detailed in Figure 15.

In our previous studies, a great deal of efforts have been paid to TCM-induced hepatoprotection (He et al., 2022). Firstly, a large scale of dataset, including 677 hepatoprotective phytoconstituents and 205 hepatoprotective TCMs, was established by conducting a comprehensive literature retrieval. Then molecular network technique was adopted to construct a hepatoprotective “TCM-ingredient” network. In addition, by incorporating the use of multiple machine learning algorithms, an *in silico* model for predicting the liver protecting activity of phytoconstituents was developed for the first time. All of these studies mentioned above laid a foundation for identifying the material basis of TCM-induced hepatoprotection. In present study, we attempted to establish a computational pharmacology method to identify hepatoprotective ingredients from TCMs by integrating our previous studies. Then, the proposed method was utilized to explore the material basis and mechanism of BS against NAFLD by combining with network pharmacology, molecular docking, and molecular dynamics simulation techniques. We hope this work would be helpful to understand the scientific connotation of Bai Shao on NAFLD in a more precise manner. The detailed research project was displayed in Figure 1.

## 2 Results

### 2.1 Candidate compounds of BS

A total of 97 unduplicated compounds were extracted from TCMSP, ETCM, and TCMID databases. After investigating the pharmacokinetic parameters of DL and OB, twenty-three compounds were found to satisfy the inclusion criteria (Table 1, Compound 5—Compound 27). These compounds were defined as candidate compounds and further analyzed.

**TABLE 1:** Potential hepatoprotective ingredients in BS.

ID	Compound (structural classification) (DL, OB)	2D structure	Literature verfication	Potential hepatoprotective ingredients		
				M1	M2	M3
1	Gallic Acid (Phenolic acids)	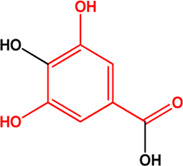	Girish and Pradhan (2012)	**√**		
2	Astragalin (Flavonoids)	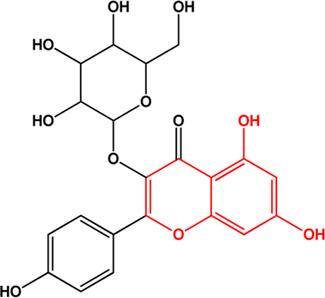	Ghosh et al. (2011)	**√**		
3	Oleanolic Acid (Triterpenes)	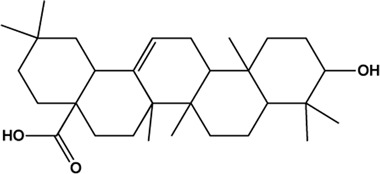	(Girish and Pradhan, 2012)	**√**		
4	Betulinic Acid (Triterpenes)	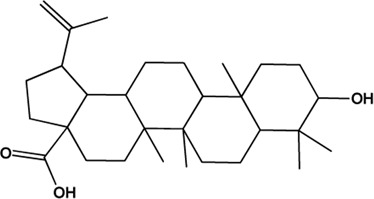	Yao et al. (2016)	**√**		
5	Benzoylpaeoniflorin (Monoterpene glucosides) (0.54, 31.14%)	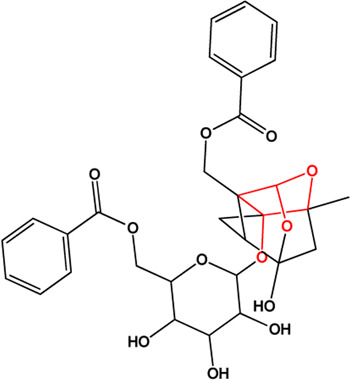	Kaur et al. (2018)	**√**	**√**	
6	Paeonol (Phenolic acids) (Good, 0)	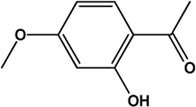	Hu et al. (2016)	**√**	**√**	
7	Kaempferol (Flavonoids) (0.24, 41.88%)	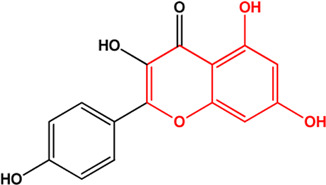	Hu et al. (2016)	**√**	**√**	
8	Paeoniflorin (Monoterpene glucosides) (0.79, 53.87%)	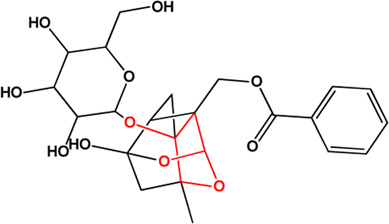	Kaur et al. (2018)	**√**	**√**	
9	Albiflorin (Monoterpene glucosides) (0.77, 30.25%)	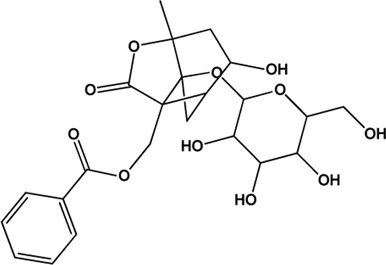	Jeong et al. (2017)	**√**	**√**	
10	Progallin A (Phenolic acids) (Moderate, 0)	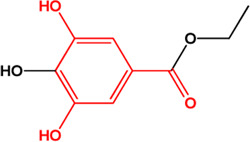	Ezhilarasan et al. (2024)	**√**		
11	(+)-Catechin (Flavonoids) (0.24, 54.83%)	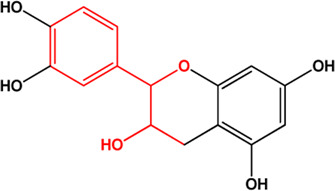	(Girish and Pradhan, 2012)	**√**		
12	β-Sitosterol (Triterpenes) (0.75, 36.91%)	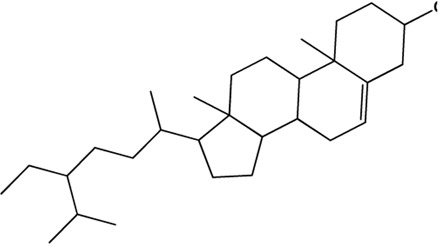	(Girish and Pradhan, 2012)	**√**		
13	Epigallocatechin (Flavonoids) (Moderate, 0)	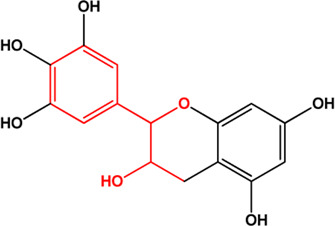	Musial et al. (2020)		**√**	
14	Oxypeucedanin (Coumarins) (Moderate, 0)	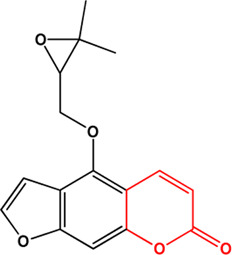	Oh et al. (2002), Park et al. (2020)			**√**
15	Benzoic acid (Phenolic acids) (Moderate, 0)	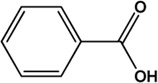	Van Puyvelde et al. (1989)			**√**
16	α-Cedrene (Volatile oils) (Moderate, 0)	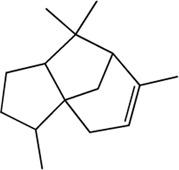	Vinholes et al. (2014)			**√**
17	Palbinone (Monoterpene glucosides) (0.53, 43.56%)	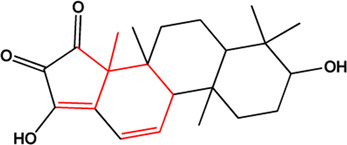	Li et al. (2023)			**√**
18	Paeonoside (Phenolic acids) (Moderate, 1)	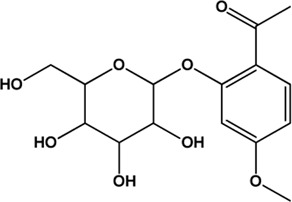	Wang et al. (2023b)			**√**
19	Lactiflorin (Monoterpene glucosides) (0.80, 49.12%)	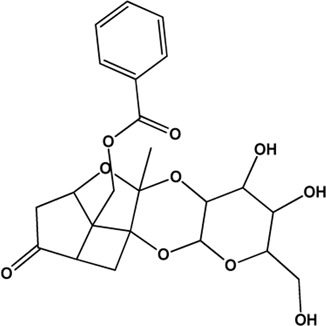	No report			**√**
20	11alpha,12alpha-epoxy-3beta-23-dihydroxy-30-norolean-20-en-28,12beta-olide (Triterpenes) (0.38, 64.77%)	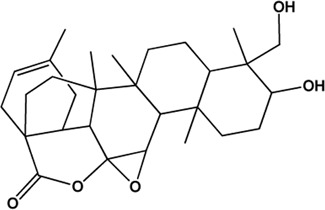	No report			**√**
21	Eugenitin (Naphthalenones) (Good, 0)	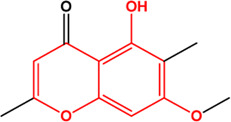	No report			**√**
22	α-Cedrol (Volatile oils) (Moderate, 0)	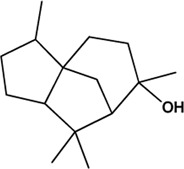	Chang et al. (2020)			**√**
23	1-Hydroxydodecane (Alcohols) (Moderate, 0)	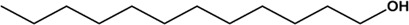	No report			**√**
24	Paeoniflorigenone (Monoterpenes) (0.37, 87.59%)	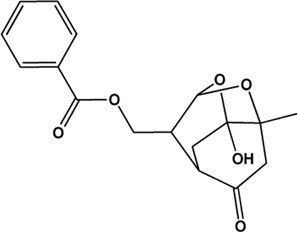	No report			**×**
25	Paeonilactone A (Monoterpenes) (Moderate, 0)	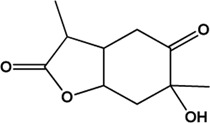	No report			**×**
26	(+)-Trans-myrtanol (Volatile oils) (Moderate, 0)	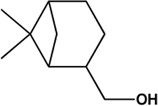	No report			**×**
27	Paeonilactone C (Monoterpenes) (Good, 0)	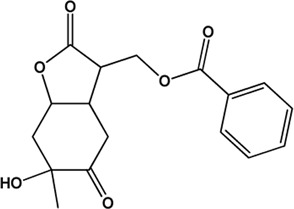	No report			**×**

M1, M2, and M3 were the abbreviations of module 1, module 2, and module 3, respectively. The potential hepatoprotective ingredients identified by each module were marked with “√,” and the unique hepatoprotective ingredients identified by each module were highlighted in red. In addition, those compounds which were predicted as non-hepatoprotective ingredients by module three were marked with “×.” The substructures highlighted in red were the representative substructures for hepatoprotective activity.

### 2.2 Identification of the potential hepatoprotective ingredients in BS

As shown in Table 1, a total of 23 potential hepatoprotective ingredients (Compound 1—Compound 23) were identified by the computational pharmacology method proposed. Module 1, module 2, and module 3 separately identified 12, 6, and 10 potential hepatoprotective ingredients. The unique hepatoprotective ingredients provided by these three modules were 7, 1, and 10 in number, respectively. Such a phenomenon indicated that there existed complementary effects among module 1, module 2, and module 3. In other words, it was necessary to adopt a multi-module fusion strategy to clarify the material basis of TCM-induced hepatoprotection. In fact, we also attempted to collect the hepatoprotective ingredients in BS by retrieving concerned literature. As a result, only three hepatoprotective ingredients, including paeoniflorin (Kaur et al., 2018), albiflorin (Jeong et al., 2017), and palbinone (Li et al., 2023), were collected. Obliviously, compared with the method of retrieving literature, the computational pharmacology method proposed in the current study was able to identify the material basis of TCM-induced hepatoprotection more comprehensively.

**FIGURE 2:**
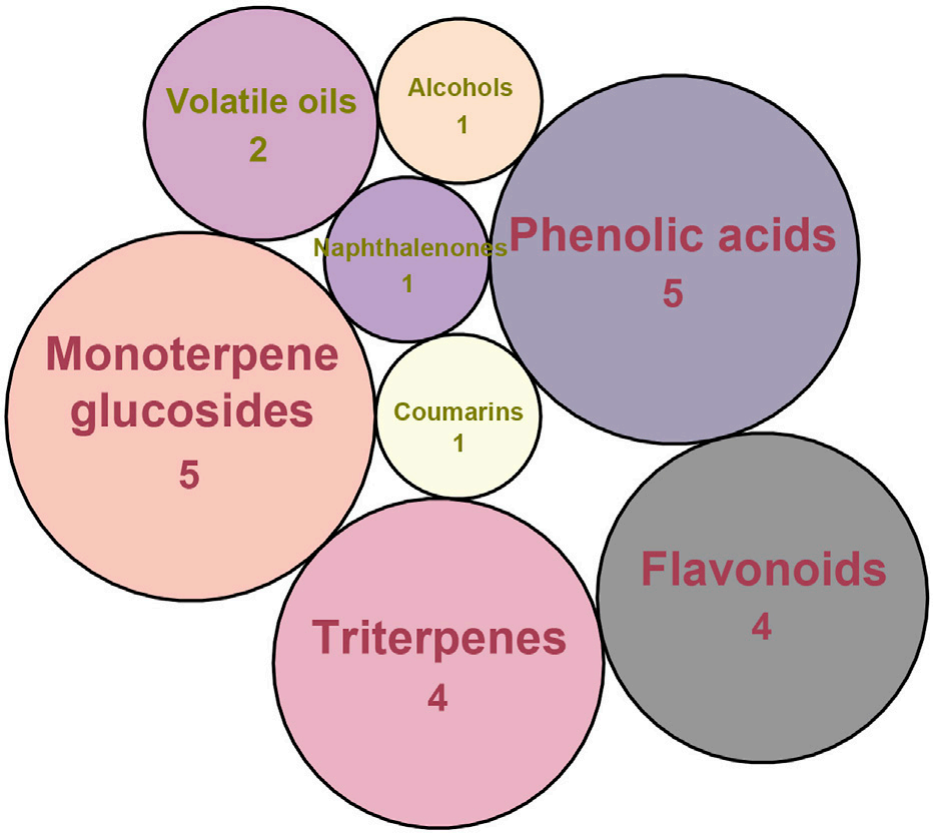
Structural classification of the potential hepatoprotective ingredients in BS.

Considering that the data included in module one and module 2 has been well documented in previous studies, the reliability of the results provided by these two modules was not discussed here. To investigate the reliability of the results provided by module 3, we have attempted to find some important evidence from the literature. As a result, among those 10 potential hepatoprotective ingredients identified by module 3, a total of six ingredients (compound 14—compound 18 and compound 22) have been confirmed to be liver-friendly substances. A range of liver-protecting activities were involved, including against chemical and drug induced liver injury (Van Puyvelde et al., 1989; Oh et al., 2002; Chang et al., 2020; Wang F. et al., 2023), anti-proliferation (Park et al., 2020), anti-lipid peroxidation (Vinholes et al., 2014), and anti-hepatic fibrosis (Li et al., 2023). For those four non-hepatoprotective ingredients (compound 24—compound 27) identified by module 3, although there was no clear evidence that they are non-hepatoprotective ingredients, no one of them was reported to be potential liver protective drugs. In addition, in our prior study (He et al., 2022), we identified a series of representative substructures (RSs) for the hepatoprotective activity of phytoconstituents. Here, structure matching between the RSs and the potential hepatoprotective ingredients was performed. In consequence, among those 10 potential hepatoprotective ingredients identified by module 3, a total of 3 (compound 14, 17, and 21) ingredients were found to contain RSs. Conversely, for those four non-hepatoprotective ingredients identified by module 3, no one of them was found to contain RSs. In summary, both the literature retrieval and the RSs matching results demonstrated that the data provided by module three was highly reliable. The structural classification of the potential hepatoprotective ingredients in BS was also investigated. As illustrated in Figure 2, monoterpene glucosides, phenolic acids, flavonoids, and triterpenes were found to be the major structure types.

### 2.3 NAFLD-related targets

**FIGURE 3:**
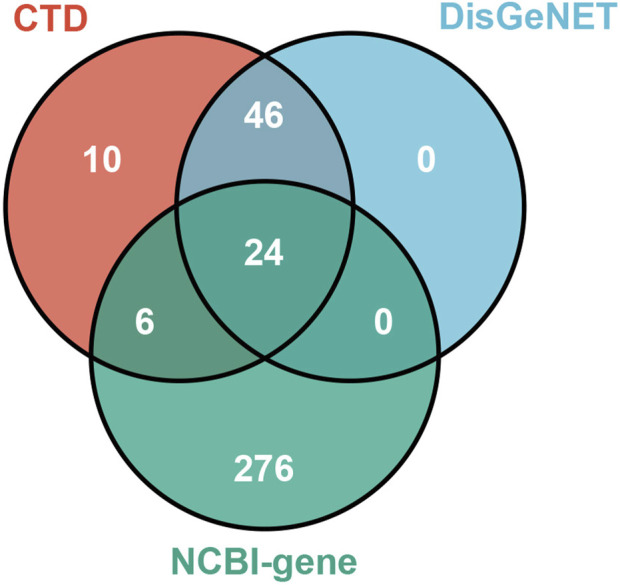
NAFLD-related targets.

As shown in Figure 3, we collected 70, 86, and 306 NAFLD-related targets from DisGeNET, CTD, and NCBI-gene database (https://www.ncbi.nlm.nih.gov/gene/), respectively. After removing the duplicates, a total of 362 NAFLD-related targets were obtained.

### 2.4 BS-related targets

By searching STITCH, CTD, Swiss Target Prediction, and DrugBank databases, we collected 773 BS-related targets. Of note, a total of eight potential hepatoprotective ingredients were found to lack of the target data, including albiflorin, oxypeucedanin, paeonoside, lactiflorin, eugenitin, α-cedrene, palbinone, and 11alpha,12alpha-epoxy-3beta-23-dihydroxy-30-norolean-20-en-28,12beta-olide. For the other 15 potential hepatoprotective ingredients, the number of targets ranged between 1 and 501.

### 2.5 “Ingredient-target” network of BS on NAFLD

**FIGURE 4:**
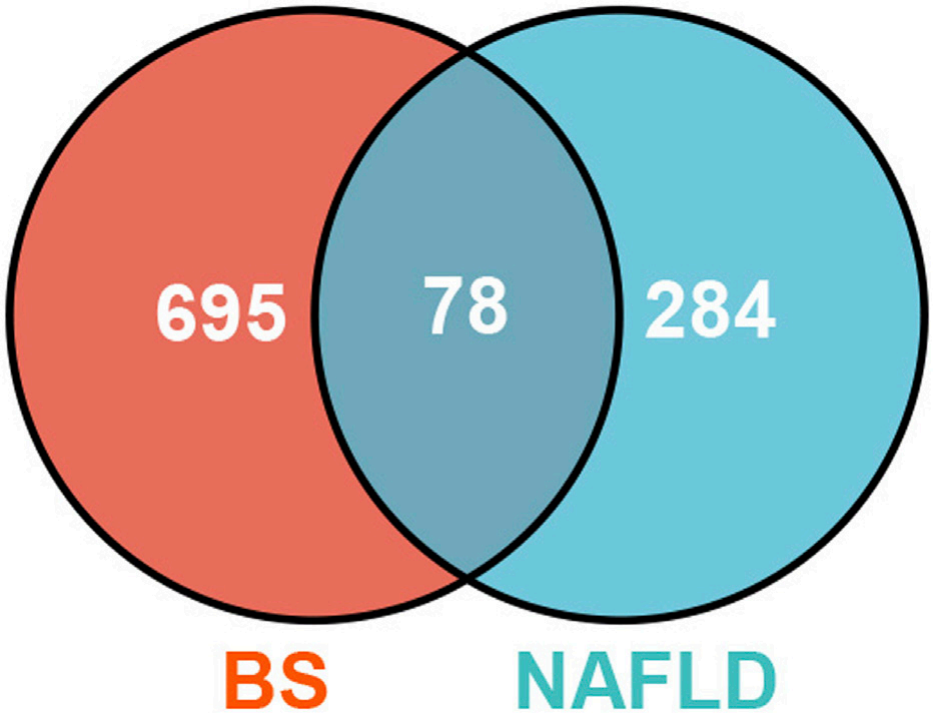
The common targets between BS and NAFLD.

**FIGURE 5:**
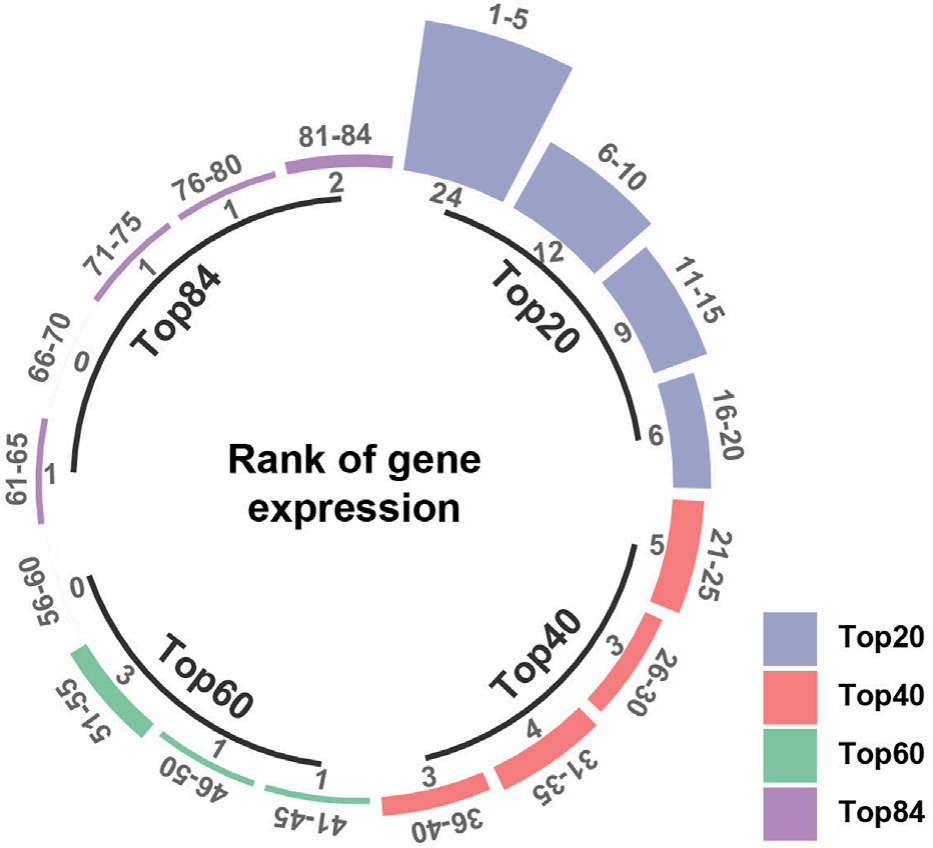
Tissue-specific expression pattern analysis of the common targets between BS and NAFLD. If a gene expressed highest in the liver among the 84 organs, it was ranked 1. Inversely, if it expressed lowest in the liver, it was ranked 84.

**FIGURE 6:**
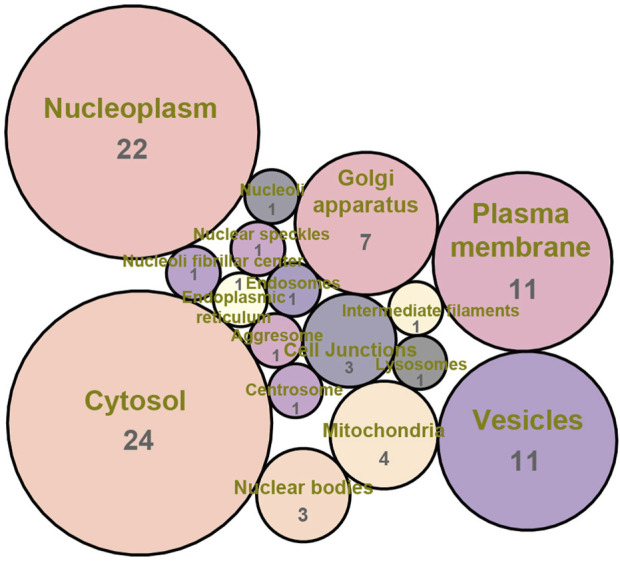
Subcellular localization analysis of the common targets between BS and NAFLD.

Firstly, Venn diagram analysis was performed to identify the shared targets between BS and NAFLD. As demonstrated in Figure 4, a total of 78 common targets were identified. Tissue-specific expression pattern analysis showed that there were 36 targets that have the liver expression levels ranked in the top 10 among the 84 organs of human. Fifty-one targets have the liver expression levels ranked among the top 20 (Figure 5). In summary, most of the shared targets were highly expressed in the liver tissue, which may lay a foundation for BS alleviate NAFLD. To further explore the potential biological function of the common targets, subcellular localization analysis was performed. As shown in Figure 6, the common targets were enriched in a variety of cellular compartments, and the top five cellular compartments were nucleoplasm, cytosol, vesicles, plasma membrane, and golgi apparatus, respectively. In fact, all of these five cellular compartments have been demonstrated to be closely related to the development of NAFLD (Qiu et al., 2013; Wang Y. et al., 2023; Lu et al., 2023; Sherman et al., 2023). For example, Lipin proteins, including Lipin1, Lipin2, and Lipin3, play crucial roles in lipid metabolism. It has been reported that Lipin3 heterozygous knockout mice was more easily to suffered from NAFLD. Further mechanistic study suggested that such a situation was associated with the abnormal distribution of Lipin1 in cytosol and nucleoplasm (Wang F. et al., 2023).

**FIGURE 7:**
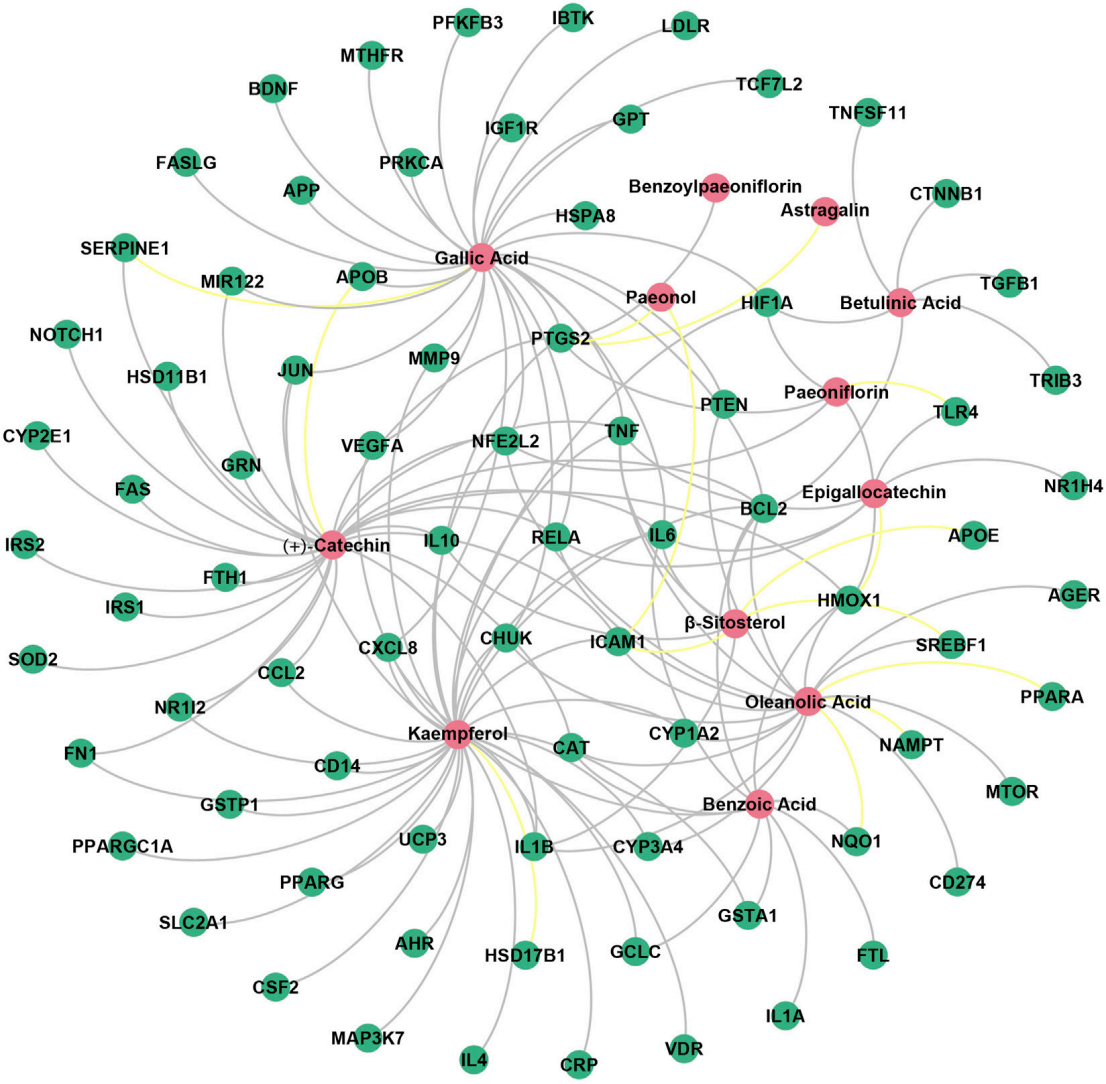
“Ingredient-target” network of BS on NAFLD. The red nodes represented the potential hepatoprotective ingredients in BS, and the green nodes represented the NAFLD-related targets. In addition, the known targets and the putative targets were connected with the potential hepatoprotective ingredients by grey and yellow edges, respectively.

Finally, the “ingredient-target” network of BS on NAFLD was constructed by inputting the common targets and the common targets related compounds into the network visualization tool Gephi (version 0.9.2). As shown in Figure 7, the network consisted of 90 nodes and 147 edges. A total of 12 potential hepatoprotective ingredients were involved, including four flavonoids (kaempferol, (+)-catechin, epigallocatechin, and astragalin), three triterpenes (oleanolic acid, β-sitosterol, and betulinic acid), three phenolic acids (gallic acid, benzoic acid, and paeonol), and two monoterpene glucosides (paeoniflorin and benzoylpaeoniflorin). The anti-NAFLD effect of BS may be largely attributed to these ingredients. In addition, a total of 78 NAFLD-related targets were included in the “ingredient-target” network. To distinguish the known targets from the putative targets, we highlighted the edges by grey and yellow, respectively. It is not difficult to find that more than 90% of the edges were showed in grey, indicating that the “ingredient-target” network developed has a high reliability.

### 2.6 PPI network analysis

**FIGURE 8:**
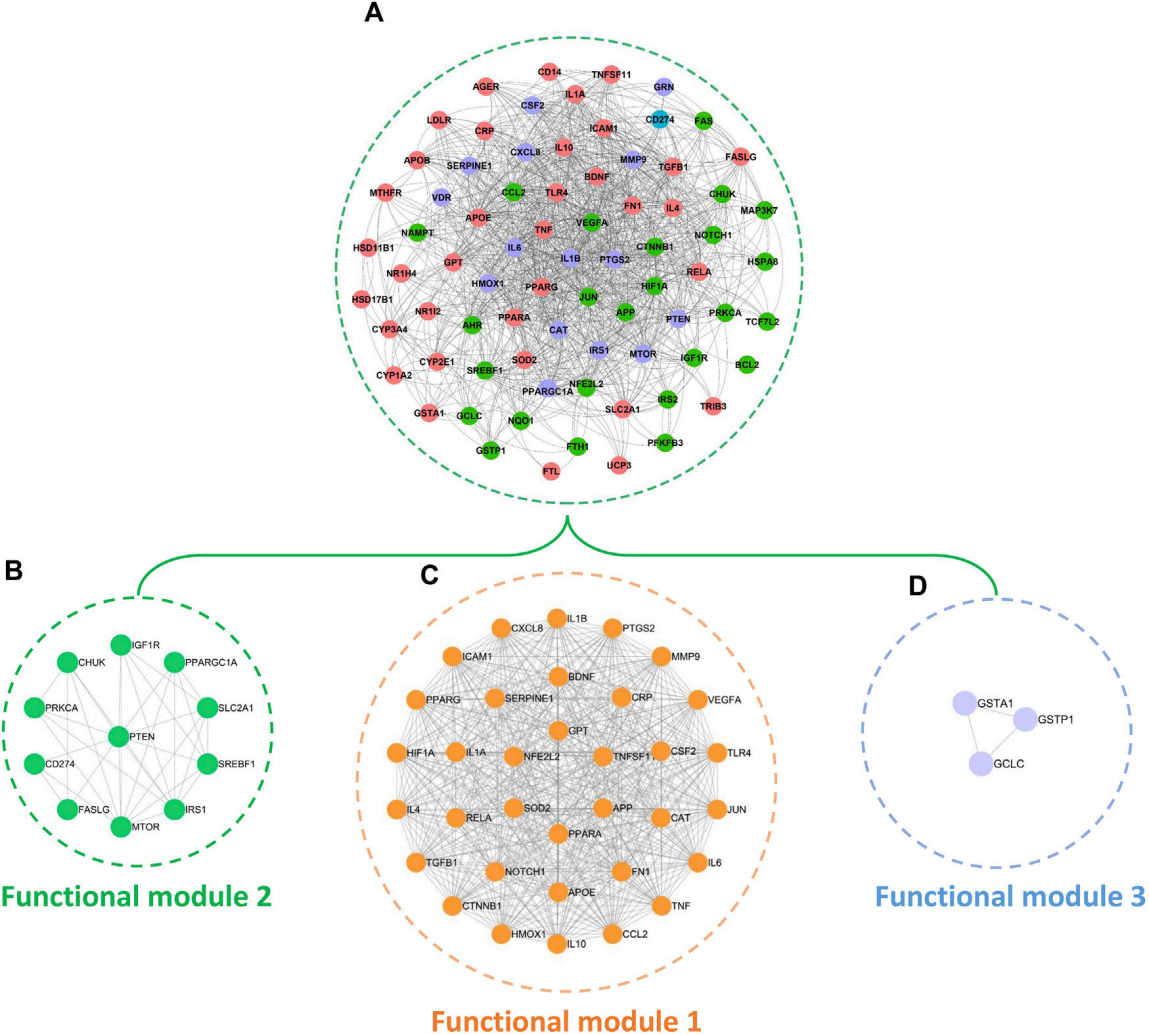
PPI network **(A)** and functional modules **(B–D)** of BS on NAFLD.

By mapping the 78 common targets identified in Section 2.5 into the STRING database, we acquired the PPI network of BS on NAFLD (Figures 8A). This network consisted of 76 nodes and 1,111 edges. The red, purple, and green nodes represented the targets that have the liver expression levels ranked in the top 10, top 20, and top 84 among the 84 organs of human, respectively. Of note, the tissue-specific expression data of CD274 was unavailable. Therefore, here, we highlighted it by blue. Modular analysis found that the PPI network can be divided into three functional modules (Figure 8B–D). As the major functional module of BS on NAFLD, functional module one consisted of 34 nodes and 494 edges. Functional module three is the minimal functional module which consisted of three nodes and three edges. In addition, functional module two including 11 nodes and 32 edges.

### 2.7 KEGG pathway enrichment analysis

In the previous section, we identified three functional modules of BS on NAFLD. Herein, by mapping the targets included in each functional module into the DAVID platform, we obtained 23 NAFLD-related KEGG pathways in total. All of these 23 pathways have been well documented to be associated with the development of NAFLD.

**FIGURE 9:**
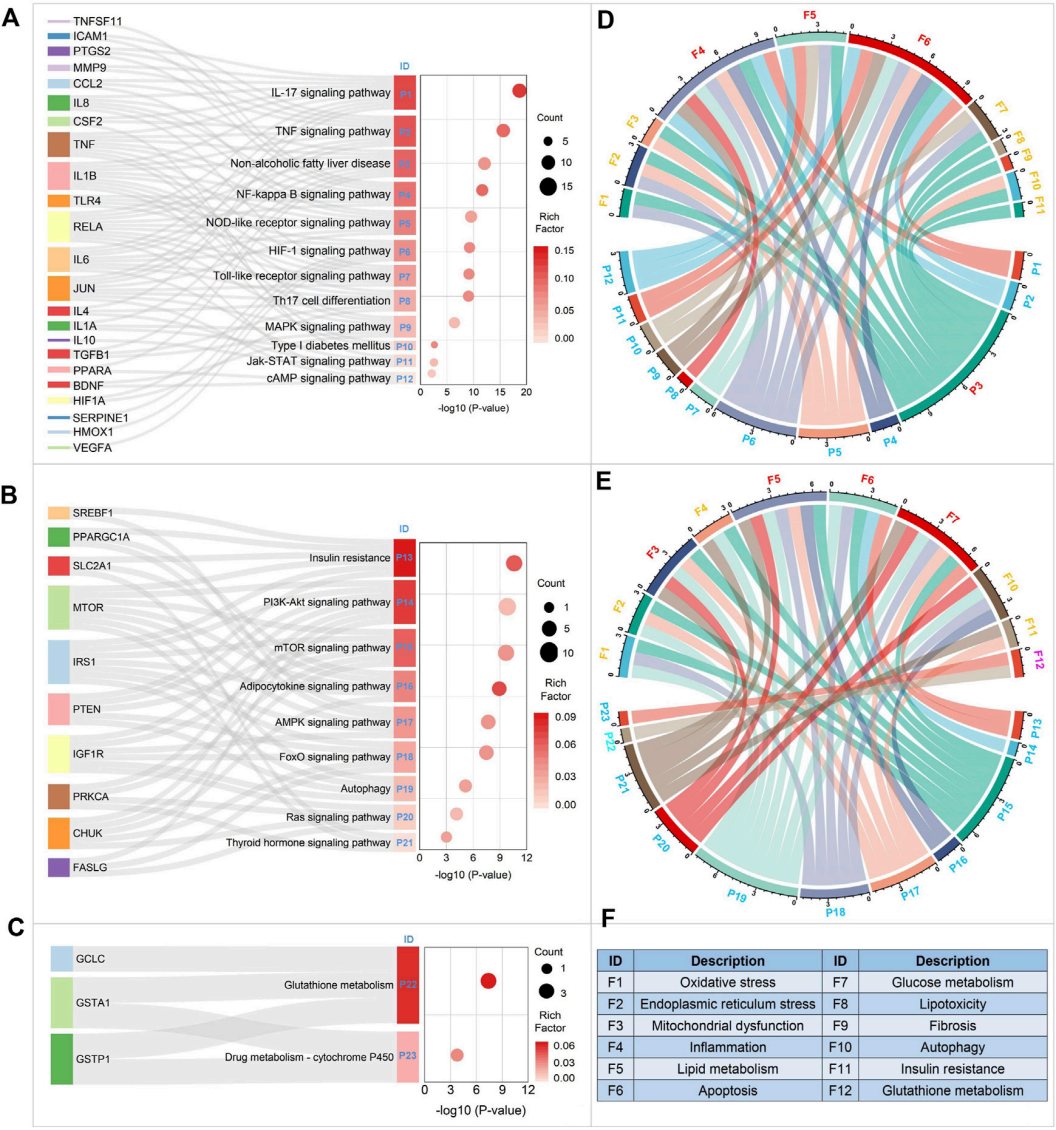
KEGG pathway enrichment analysis of the major functional modules of BS on NAFLD. Figures **(A–C)** displayed the KEGG pathways enriched by functional modules 1, 2, and 3, respectively. In Figure **(F)**, F1-F12 were the major biological processes which were involved in the occurrence and development of NAFLD. Figure **(D)** displayed the NAFLD-related biological processes enriched by functional module 1. The NAFLD-related biological processes enriched by functional module two and three were showed in Figure **(E)**.

As shown in Figure 9A–C, a total of 12 KEGG pathways were enriched by functional module 1. Functional module two and functional module 3 separately enriched nine and 2 KEGG pathways. Then we sorted these pathways based on the adjusted *p*-value. In consequence, we found that the top five pathways were IL-17 signaling pathway, TNF signaling pathway, Non-alcoholic fatty liver disease, NF-kappa B signaling pathway, and Insulin resistance, respectively. Interestingly, the pathway of Non-alcoholic fatty liver disease was enriched significantly with the adjusted *p*-value ranked third, confirming the moderating effect of BS on NAFLD to a great extent. In addition, it’s worth noting that all of the top four pathways were enriched by functional module 1, suggesting that it may be the major functional module of BS on NAFLD.

To elucidate the regulation effects of those three functional modules on NAFLD from the perspective of biological process. Firstly, we summarized the major biological processes associated with NAFLD by conducting a systematical literature retrieval. As showed in Figure 9F, we collected 12 NAFLD-related biological processes in total. Then, for each KEGG pathway enriched above, the related biological processes were annotated based on KEGG pathway database. In addition, we also retrieved the scientific literature database, by which we attempted to obtain the latest and most complete biological process data. Finally, Chord Plot was plotted to display the interactions between the KEGG pathways and the NAFLD-related biological processes (Figure 9E,F). As a result, we found that the major biological processes regulated by functional module one were inflammation, apoptosis, and lipid metabolism. For functional module 2, a total of four major NAFLD-related biological processes were observed, including glucose metabolism, lipid metabolism, mitochondrial dysfunction, and apoptosis. Functional module three was found to involve in the biological process of glutathione metabolism. It is not difficult to found that these three functional modules regulated NAFLD through different biological processes in a synergetic manner.

**FIGURE 10:**
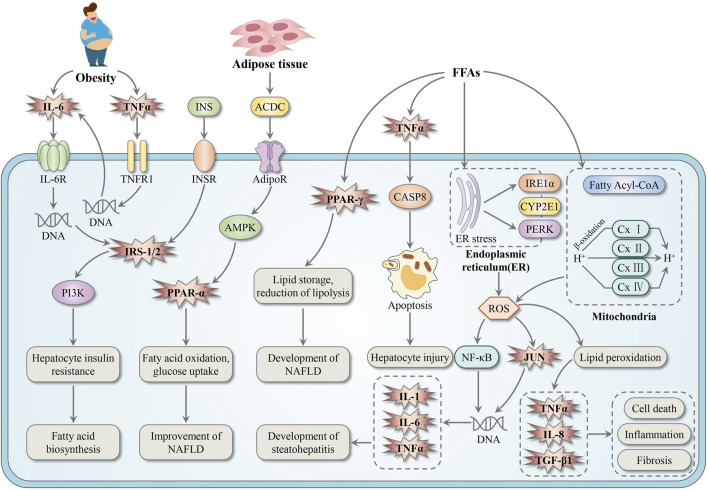
The mechanism map of BS relieve NAFLD. The genes regulated by BS were highlighted in pink explosive shape. FFAs, free fatty acids.

By mapping the BS-related targets into the Non-alcoholic fatty liver disease pathway, we attained a detailed mechanism map of BS against NAFLD. As illustrated in Figure 10, BS may relieve NAFLD through three major paths listed as follows. Firstly, it’s well known that abnormal glucose and lipid metabolism was one of the major inducements of NAFLD (Chao et al., 2019). IRS-1/2, PPAR-α, and PPAR-γ have been proved to be essential regulatory factors of glucose and lipid metabolism (Chao et al., 2019). Therefore, ameliorating glucose and lipid metabolism via regulating IRS-1/2, PPAR-α, and PPAR-γ may be one of the major mechanisms of BS on NAFLD. Secondly, as one promising therapeutic strategy of NAFLD, decreasing hepatocyte apoptosis has gained increasing attention in recent years (Canbay et al., 2005). The significant positive regulatory effect of TNF-α on hepatocyte apoptosis has been well documented by many pharmacologists (Ezquerro et al., 2019). Thus, inhibiting the pro-apoptosis effect of TNF-α and decreasing hepatocyte injury may be another important mechanism of BS on NAFLD. Thirdly, inflammation is an important pathological change from steatosis to hepatitis (Patel and Mueller, 2024). This pathological change was significantly associated with the increase of expression levels of pro-inflammatory cytokines and inflammatory cytokines (Rao et al., 2018). Therefore, inhibiting the overexpression of IL-1, IL-6, IL-8 and TNF-α may be the third path of BS on NAFLD. In addition, it has been reported that TGFβ1 is a powerful fibrogenic cytokine (He et al., 2016). Depending on the data displayed in Figure 10, we speculated that some ingredients in BS may be TGFβ1 agonists which can alleviate liver fibrosis to a certain extent. In summary, we believed that the anti-NAFLD effect of BS should be attributed to multi-target, multi-pathway, and multi-biological process.

In addition, we investigated the reliability of the mechanisms of BS on NAFLD attained above via a literature-based method. Firstly, we summarized the molecular mechanisms of BS extracts induced hepatoprotection by conducting a systemic literature retrieval (Table 2). After that, consistency estimate between our results and the literature reports was performed. As a result, in the level of pathway, among those 23 pathways enriched by the DAVID platform, at least 11 pathways have been proved to be associated with BS-induced hepatoprotection, including P4, P6, P7, P11, P12, P13, P14, P15, P17, P18, and P22. Besides, at least five pathways (P4, P7, P13, P14, and P17) were reported to play essential roles in the process of BS alleviates NAFLD. In the level of biological process, among those 12 biological processes identified in Figure 9D,E, at least eight biological processes (F1, F3, F4, F5, F6, F7, F9, and F11) have been demonstrated to be significantly associated with the anti-NAFLD effect of BS. In the level of gene, among the pathway of NAFLD, a total of nine genes were found to be the potential targets of BS (Figure 10). According to the available literature, we found that eight out of these nine genes can be significantly regulated by BS, including IL-6, TNF-α, IRS1, PPAR-γ, IL-1β, JUN, TGF-β1, and PPAR-α (Table 2). To be specific, PPAR-α can be upregulated by BS, and the other seven genes can be downregulated by BS. All of above mentioned indicated that our results were highly in accord with the data recorded in the literature, suggesting a higher reliability of our method.

**TABLE 2:** Molecular mechanisms of BS extracts-induced hepatoprotection.

Liver disease	BS extracts	Pathway	Biological process	Gene expression	References
NAFLD	TGP	–	F5, F11	–	Zheng et al. (2008)
		–	F3	–	Sun et al. (2022)
		–	F5, F11	–	Zheng et al. (2008)
		P7	F5, F7, F11	–	Yang et al. (2017)
		–	F1, F4, F5, F6	IL-6↓, TNF-α↓	Ji (2022)
	Paeoniflorin	–	F4, F5, F7, F11	PPAR-α↑, PPAR-γ↓, TNF-α↓, IL-1↓, IL-6↓	Zhang et al. (2015)
		P13, P14	F5, F11	IRS1↓	Ma et al. (2017)
		P14, P17	F5, F7, F11	–	Li et al. (2018)
		P17	F5, F11	–	Chen et al. (2013)
	Palbinone	P4	F4, F9	IL-1β↓, TGF-β1↓	Li et al. (2023)
Hepatic fibrosis	TGP	P12	F1, F4, F9	TNF-α↓, IL-1β↓, TGF-β1↓	Wang (2005)
	Paeoniflorin	P6, P15	F9	HIF-1α↓	Zhao et al. (2014)
		P4, P6	F4, F9	HIF-1α↓, IL-1β↓, TGF-β1↓, IL-6↓, TNF-α↓	Liu et al. (2023)
		–	F4, F9	IL-1β↓, TNF-α↓	Zhang et al. (2022b)
Cholestasis	TGP	P14	F1, F12	-	Ma et al. (2015)
	WEP	P4	F4	IL-1β↓	Ma et al. (2018)
	Paeoniflorin	P14, P22	F1, F12	–	Chen et al. (2015b)
		–	F6	–	Zhou et al. (2017)
		P4	F4	IL-1β↓	Zhao et al. (2017)
DILI	TGP	–	F1, F4, F5	TNF-α↓	Qin and Tian (2011)
		–	F4	IL-1β↓, TNF-α↓, IL-6↓	Peng et al. (2023)
	WEP	P4, P17	F4, F6	IL-6↓, TNF-α↓	Shin et al. (2022)
	Paeoniflorin	P4	F4	IL-1β↓, TNF-α↓	Chen et al. (2021)
		P18	F1, F3, F4	IL-1β↓, TNF-α↓	Li et al. (2022)
		–	F4, F6	JUN↓	Deng et al. (2022)
Cirrhosis	TGP	–	F4, F6, F9	IL-1β↓, TNF-α↓, IL-6↓	Zhang et al. (2022a)
Hepatitis	Paeoniflorin	P4, P7	F4	IL-6↓, TNF-α↓	Chen et al. (2015a)
Liver injury	Paeoniflorin	P17	F1, F4, F5	IL-1β↓, TNF-α↓, IL-6↓	Liu et al. (2022)
HCC	Paeoniflorin	P11	-	STAT3↓	Gao et al. (2023)

ID, of the pathway and biological process corresponded to the ID, in Figure 9. DILI, drug-induced liver injury; TGP, total glucosides of paeony; WEP, water extract of paeony; HCC, hepatocellular carcinoma.

### 2.8 Key targets of BS on NAFLD

**FIGURE 11:**
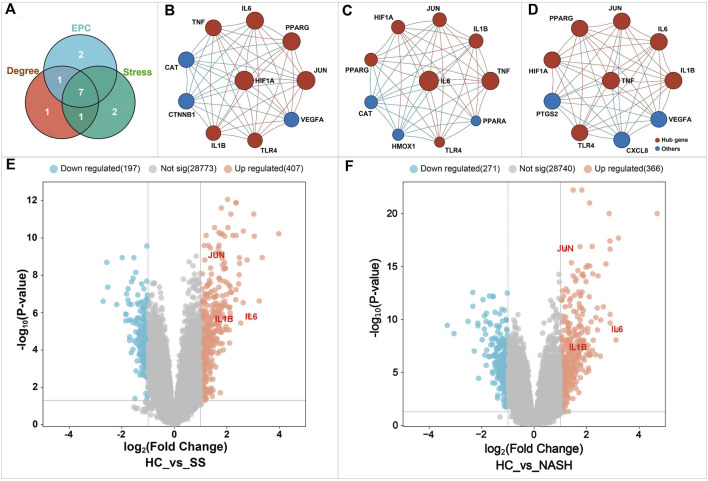
Identification of the key targets of BS on NAFLD. Figure **(A)** Venn diagram analysis. Figures **(B–D)** showed the top 10 genes in the PPI network identified by Degree, Stress, and EPC algorithms, respectively. The node size was proportional to the gene score. Higher score indicated a higher ranking. Figures **(E, F)** displayed the differentially expressed genes related to NAFLD. HC, healthy controls; SS, simple steatosis; NASH, non-alcoholic steatohepatitis.

Here, we ranked the genes in the PPI network based on CytoHubba. A total of three algorithms were adopted, including Degree, MCC, and EPC. For each algorithm, the top 10 genes were extracted, and the common genes among these three algorithms were defined as hub genes. As demonstrated in Figure 11A–D, a total of seven genes were identified as hub genes, including hypoxia-inducible factor 1-alpha (HIF1A), interleukin-1 beta (IL1-β), interleukin-6 (IL6), proto-oncogene c-Jun (JUN), peroxisome proliferator-activated receptor gamma (PPAR-γ), toll-like receptor 4 (TLR4), and tumor necrosis factor (TNF).

To further investigate the relationship between the hub genes and NAFLD, microarray data analysis was performed (Figure 11E,F). Consequently, a total of 637 differentially expressed genes were identified in the group of nonalcoholic steatohepatitis, including 271 downregulated and 366 upregulated genes. In the simple steatosis group, we observed 197 downregulated and 407 upregulated genes, respectively. These differentially expressed genes may play critical roles in the occurrence and development of NAFLD. Further analysis found that only three hub genes named IL1-β, IL6, and JUN were included in the datasets of differentially expressed gene. These three hub genes were defined as the key genes of BS on NAFLD and further analyzed. In fact, it has been demonstrated that inflammation and apoptosis were important pathogenesis of NAFLD which can be activated by the overexpression of IL1-β, IL6, and JUN (Li et al., 2013; Zhang J. et al., 2022; Deng et al., 2022). Interestingly, as shown in Table 2, the inhibitory effect of BS on these three genes has been well documented in previous studies, suggesting the importance of these three genes in the process of BS against NAFLD.

**FIGURE 12:**
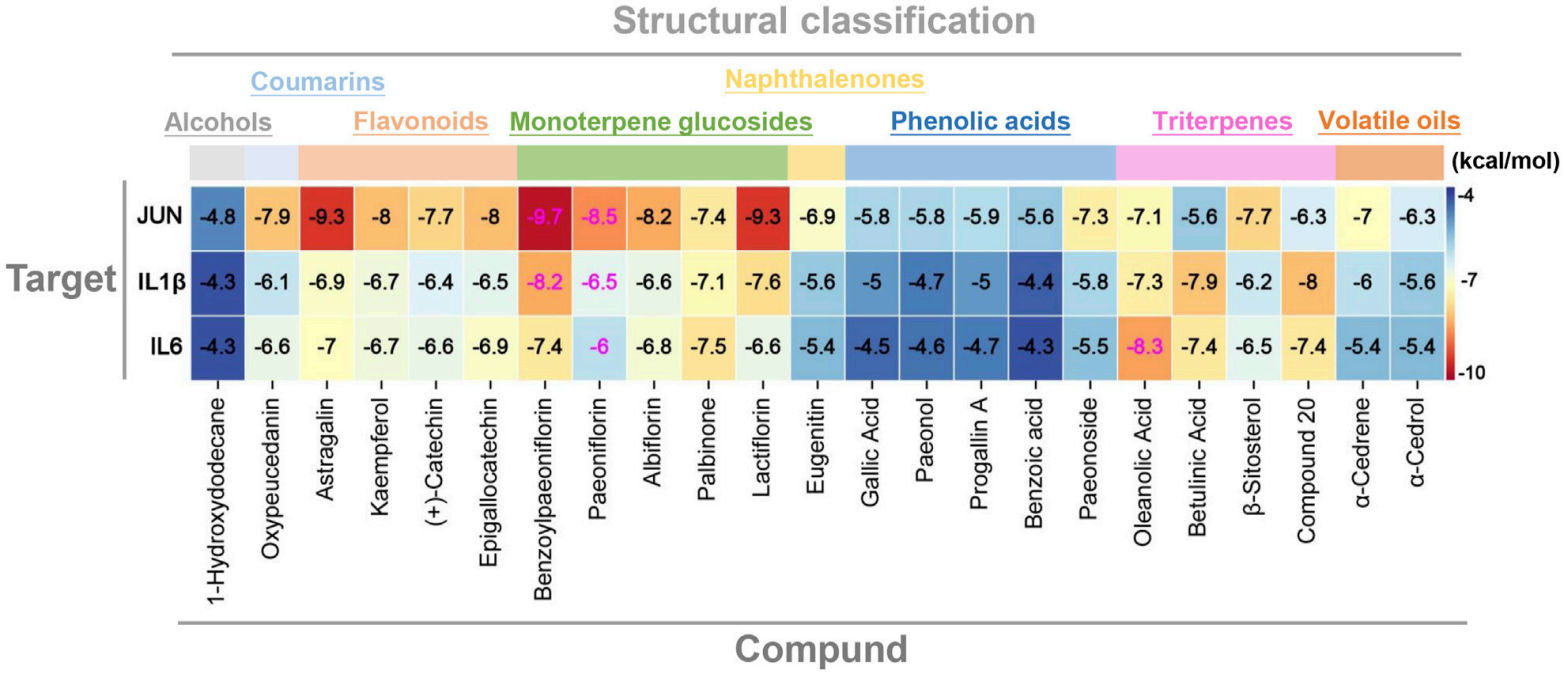
Molecular docking score of the protein–ligand complexes. Compound 20: 11alpha, 12alpha-epoxy-3beta-23-dihydroxy-30-norolean-20-en-28,12beta-olide.

To answer the question that whether there exists significant interaction between the key genes identified above and the potential hepatoprotective ingredients in BS, molecular docking analysis was carried out. Here, the index of binding energy was calculated to evaluate the binding capacity between the ligand and the receptor. The specific docking parameters were provided in Table 3. Generally, a lower value of binding energy indicates a stronger interaction between the ligand and the receptor. It has been demonstrated that there may exist significant binding capacity between the ligand and the receptor when the binding energy between them is less than −1.2 kcal/mol (Ma et al., 2022). As shown in Figure 12, the binding energies between the potential hepatoprotective ingredients and the key genes ranged from −9.7 kcal/mol to −4.3 kcal/mol. Obviously, the binding energies of all studied docking complexes were within the acceptable range. Therefore, we can claim that the hepatoprotective ingredients have great potential to bind with the key genes and affect their biological effects. Among all of the potential hepatoprotective ingredients, monoterpene glucosides, triterpenes, flavonoids, and coumarins were found to have lower values of binding energy with the key genes, indicating that there may exist stronger binding affinity between these ingredients and the key genes. In addition, compared with the genes of IL1-β and IL6, JUN exhibited stronger binding affinity with the hepatoprotective ingredients. So, we speculated that JUN may play a non-negligible role in the process of BS alleviate NAFLD.

**TABLE 3:** Molecular docking parameters.

Target	PDB ID (Resolution)	Coordinate
IL6	1ALU (1.90 Å)	x = 2.688, y = −19.9, z = 8.838
IL1β	5R8K (1.47 Å)	x = 38.561, y = 13.365, z = 68.653
JUN	3PZE (2.00 Å)	x = 15.723, y = 15.996, z = 23.498

**FIGURE 13:**
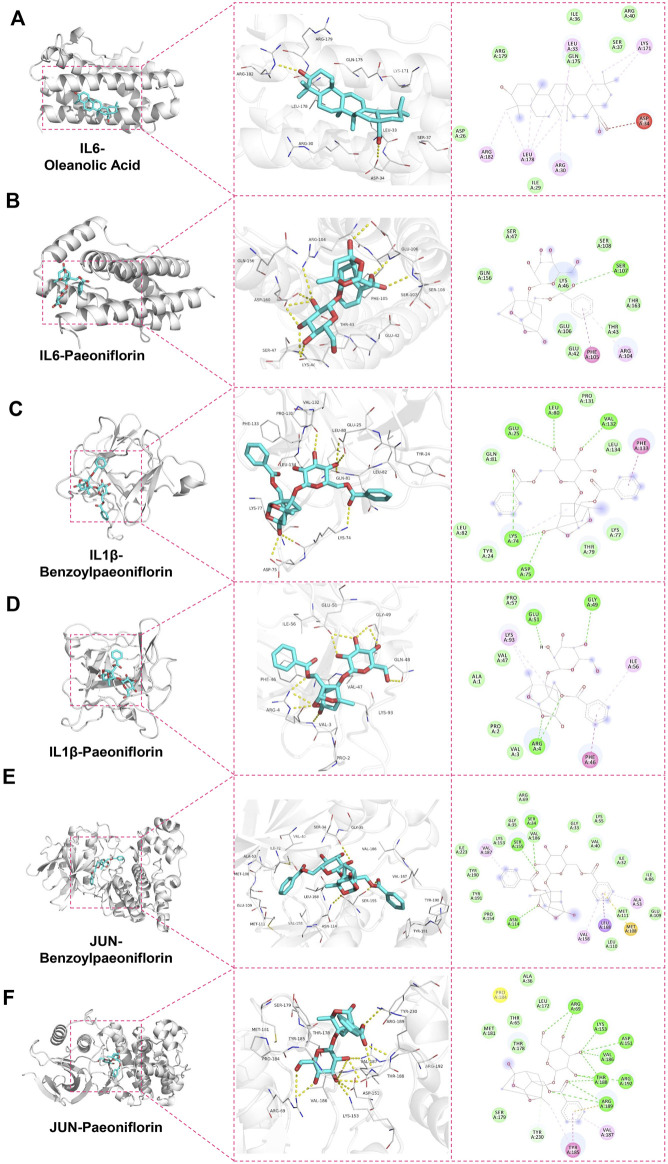
Docking modes of the protein-ligand complexes which were selected to perform MD simulation. Figures **(A, C, and E)** separately represented the docking complexes with the lowest binding energy for IL6, IL1β, and JUN. The docking complexes containing paeoniflorin were display in figures **(B, D, and F)**, respectively.

**FIGURE 14:**
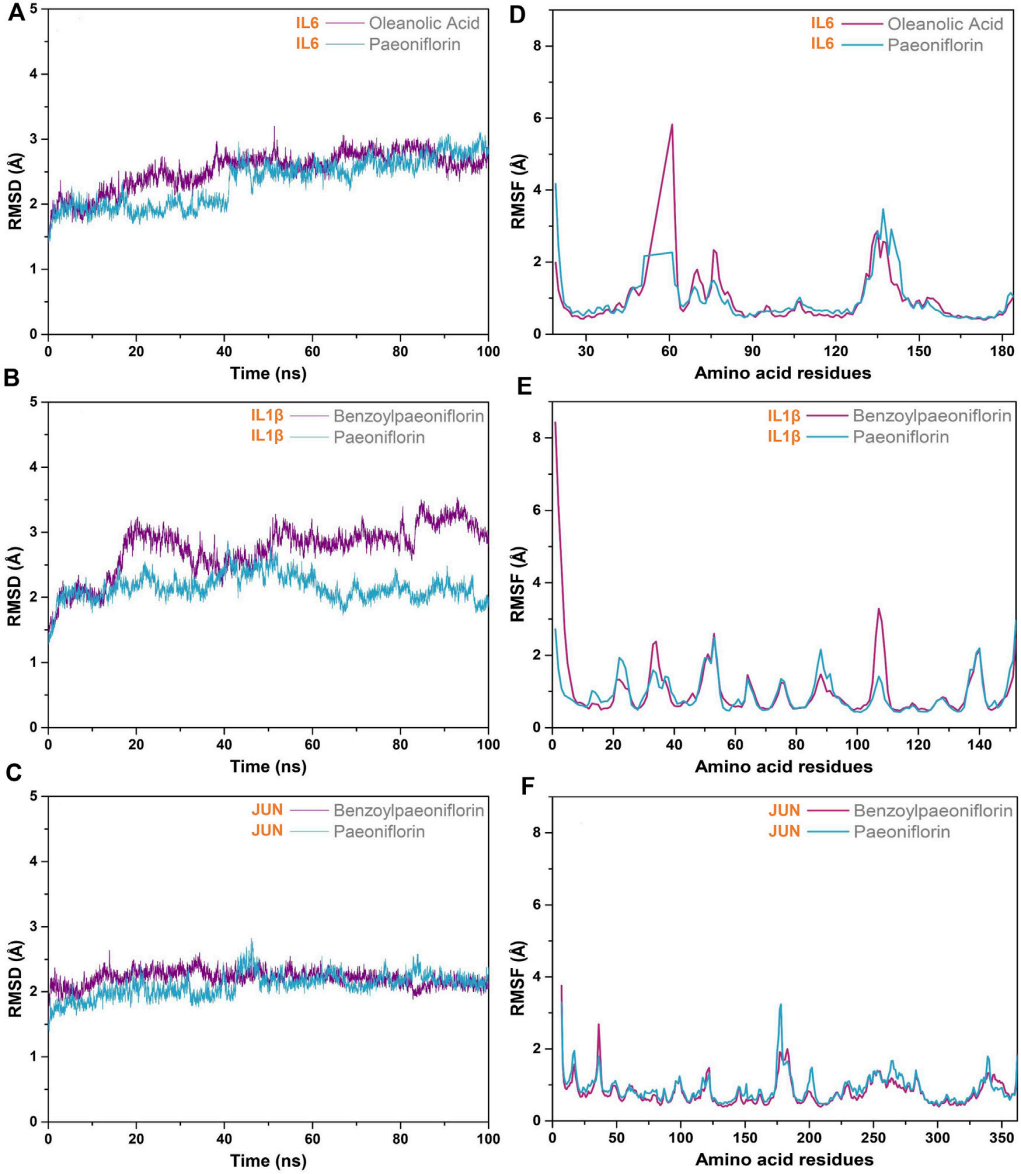
MD simulation. RMSD **(A–C)** and RMSF **(D–F)** profiles of the selected docking complexes.

The stability of the protein-ligand complexes formed by the hepatoprotective ingredients and the key genes were investigated based on the method of MD simulation. Here, for each key gene, the docking complexes with the lowest binding energy was selected to perform MD simulation (Figures 13A,C,E). Considering that paeoniflorin is the major active ingredient of BS. Therefore, the docking complexes containing paeoniflorin were also subjected to MD simulation (Figures 13B,D,F). As a result, although there existed some small fluctuations, the RMSD profiles of all studied complexes were stable in nature (Figure 14A–C)). Specifically for the complexes of JUN-benzoylpaeoniflorin and JUN-paeoniflorin, the RMSD always kept at the value of around 2 Å over the entire course of MD simulation. For the RMSF profiles (Figure 14D–F), except for the end regions and several loop regions, the RMSF values for most of the amino acid residue of the studied genes were within acceptable range with fluctuation range less than 3 Å. Furthermore, we also calculated binding free energy for each selected docking complex by utilizing the method of MMPBSA. As shown in Table 4, the binding free energy of the docking complexes were found to lie between −31.66 and −17.15 kcal/mol. In summary, all of the results of the MD simulations mentioned above demonstrated that the studied docking complexes has higher stability.

**TABLE 4:** Binding free energies of the selected docking complexes. All of the energies were provided in the format of average ± standard deviation.

ID	Docking complexes	Binding free energy (kcal/mol)
1	IL6-Oleanolic Acid	−17.15 ± 3.57
2	IL6-Paeoniflorin	−26.74 ± 3.77
3	IL1β-Benzoylpaeoniflorin	−26.01 ± 4.96
4	IL1β-Paeoniflorin	−18.99 ± 4.11
5	JUN-Benzoylpaeoniflorin	−30.77 ± 2.99
6	JUN-Paeoniflorin	−31.66 ± 4.39

## 3 Discussion

In this work, an advanced network pharmacology approach was proposed to uncover the scientific connotation of BS against NAFLD. Compared with the method of conventional network pharmacology, the research strategy adopted in the current study has the advantages of more precision. Generally, network pharmacology identifies the material basis of drugs based on the connections between the components and the disease targets, and the components exhibited higher number of disease targets connections were defined as the potential material basis. However, numerous network pharmacology research cases showed that the components connected with the disease targets were the mixtures of beneficial and harmful substances rather than the real material basis. Herein, taking TCM-induced hepatoprotection as a case, we established a novel computational pharmacology-based method to identify the material basis of TCMs, by which we attempted to improve the precision and actual application value of network pharmacology. The proposed computational pharmacology method consisted of three modules. These three modules were developed based on molecular network, database retrieval, and structure activity relationship techniques, respectively. They possess different characteristics and complement each other. Compared with the method of literature retrieval, our computational pharmacology-based method could identify the material basis of TCM-induced hepatoprotection more comprehensively and more efficiently. Based on the computational pharmacology method proposed, a total of 23 ingredients in BS were identified as liver-friendly substances, among which 19 ingredients have been demonstrated to benefit to the liver by animal or cell experiments. For the other four ingredients, although direct evidence focused on their liver-protecting effect was not found, no one of them was implicated by drug-induced liver injury. In addition, RSs analysis found that there existed abundant hepatoprotection-related RSs among the potential hepatoprotective ingredients identified. Data mentioned above indicated that our computational pharmacology method was highly reliable. We believed that our method would provide a strong technical support to disclose the material basis of TCM-induced hepatoprotection. Then based on the hepatoprotective ingredients discovered above, we investigated the material basis and mechanism of BS against NAFLD comprehensively.

Consistency analysis between the NAFLD-related targets and BS-related targets identified 78 common targets. Both the tissue-specific expression pattern analysis and subcellular localization analysis indicated that these common targets were significantly correlated with NAFLD. Molecular network analysis found that at least 12 ingredients in BS could regulate these common targets. Flavonoids, triterpenes, monoterpene glucosides, and phenolic acids were found to be the major structure types. Our prior studies suggested that phenolic acids, flavonoids, and triterpenes were important natural sources of liver protectants (He et al., 2022). In addition, monoterpene glucosides were reported to be the major active components of BS (Jiang et al., 2020). Therefore, we speculated that these 12 ingredients may be the major material basis of BS against NAFLD. Interestingly, it seemed that molecular docking analysis also verified such a speculation. Compared with the other structure types, flavonoids, triterpenes, monoterpene glucosides exhibited stronger binding affinity with the key targets of BS on NAFLD. Obviously, molecular docking analysis further indicating the importance of these ingredients in the process of BS against NAFLD. In fact, oxypeucedanin, eugenitin, α-cedrene and α-cedrol also showed stronger binding affinity with the key targets. We speculated that the lack of target data may be an important reason for these ingredients escaped from the identification of molecular network analysis. After all, for these four ingredients, only two targets were collected.

Focused on the PPI network of BS on NAFLD, modular analysis identified three functional modules in total. By mapping the targets included in each functional module into the DAVID platform, a total of 23 NAFLD-related KEGG pathways were enriched, among which not less than 11 pathways have been reported to be associated with BS-induced hepatoprotection. Besides, at least five pathways have been demonstrated to play essential roles in the process of BS alleviates NAFLD. Further analysis found that a total of 12 NAFLD-related biological processes were involved by the KEGG pathways enriched. It’s worth noting that not less than eight out of these biological processes have been proved to be involved in the process of BS against NAFLD. Except for identifying the key pathways and the major biological processes, we also identified the key targets based on hub gene analysis and microarray data analysis. As a result, a total of three genes, including IL1-β, IL6, and JUN, were identified. Through retrieving previously published literature, we found that these three key genes indeed can be significantly downregulated by BS.

In summary, our findings were highly consistent with reports in the literature, indicating that the bioinformatics strategy adopted in the current study were reasonable and reliable. However, we must acknowledge that there still existed some limitations in our research. Although we have validated our results through molecular docking analysis, molecular dynamics simulation analysis and literature analysis, but more in-depth experimental verification is required to further confirm our findings. In addition, the incomplete and missing of BS-related targets may also affect our results. After all, some potential hepatoprotective ingredients were found to lack of the target data when we collected BS-related targets. With the constant perfection of relevant data in the future, we believed that our bioinformatics strategy would achieve a more satisfactory performance.

## 4 Materials and methods

### 4.1 Collection of candidate compounds of BS

The chemical components of BS were extracted from three typical TCM databases, including TCMSP (Ru et al., 2014), TCMID (Xue et al., 2013), and, ETCM (Xu et al., 2019). The duplicates and the compounds without structures were removed. For the remaining components, the pharmacokinetics parameters, including drug likeness (DL) and oral bioavailability (OB), were investigated. Those components satisfy at least one of the following two conditions were defined as candidate compounds: (1) OB ≥ 30%, DL ≥ 0.18 (data from TCMSP database), (2) both QED (quantitative estimate of DL) and OB are good or moderate classes (data from, ETCM database).

### 4.2 A computational pharmacology method to identify the material basis of TCM-induced hepatoprotection

In this section, a computational pharmacology method was proposed to screen hepatoprotective ingredients from TCMs, by which we attempted to provide valuable clues for elucidating the material basis of TCM-induced hepatoprotection. The computational pharmacology method consisted of three modules detailed as follows.

Module 1: Module one is a hepatoprotective “TCM-ingredient” network which consisted of 638 nodes and 2,262 edges. A total of 433 hepatoprotective ingredients and 205 hepatoprotective TCMs were involved. This network intuitively displayed which compounds were associated with the liver protection of the TCMs existed in the network.

Module 2: Module two is a large scale dataset of TCM-induced hepatoprotection, including 677 hepatoprotective phytoconstituents. For each phytoconstituent, we provided the English name and the simplified molecular input line entry system (SMILES) information, which enables researchers to retrieve this dataset by the method of name or structure matching.

Module 3: Module three is an *in silico* model which aimed at predicting the hepatoprotective activity of phytoconstituents derived from TCMs. This *in silico* model was established based on 709 phytoconstituents by integrating seven types of machine learning algorithms. Both the 5-fold cross-validation and the external validation produced accuracy greater than 85%, indicating that this model is reasonably successful.

**FIGURE 15:**
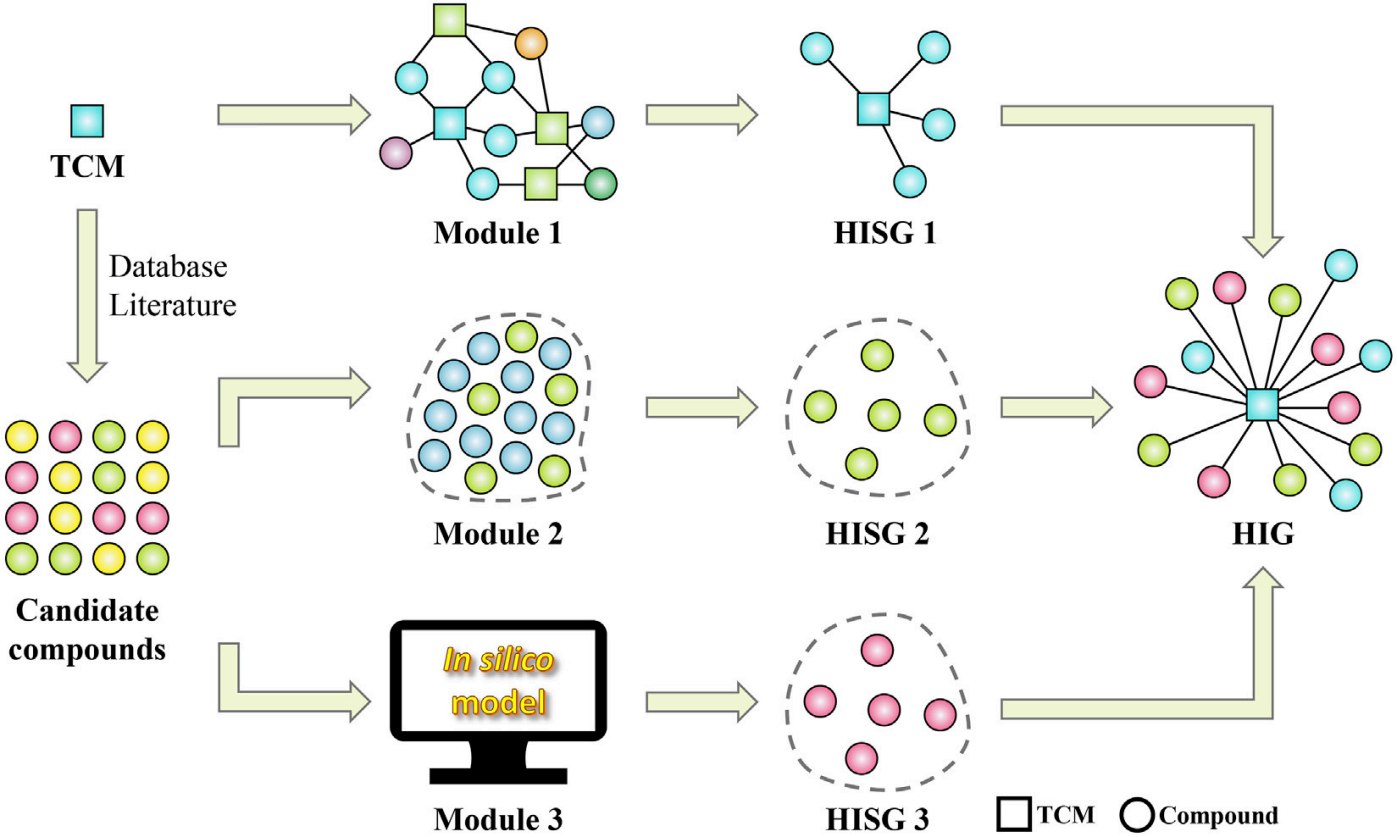
Workflow of the computational pharmacology method for identifying the material basis of TCM-induced hepatoprotection. HISG, hepatoprotective ingredient subgroup; HIG, hepatoprotective ingredient group.

Details on these three modules mentioned above can be found in our prior published work (He et al., 2022). It’s worth noting that the hepatoprotective ingredients identified by the computational pharmacology method can be classified into two categories, including the known hepatoprotective ingredients and the putative hepatoprotective ingredients. The former was provided by module one and module 2, and the latter was provided by module 3. They constitute together the material basis of TCM-induced hepatoprotection. The detailed workflow of the computational pharmacology method was provided in Figure 15.

### 4.3 Collection of targets for the potential hepatoprotective ingredients in BS

For each ingredient, the targets consisted of the known targets and the putative targets. We obtained the known targets by searching DrugBank (Wishart et al., 2008) and CTD (Wishart et al., 2008) databases using the ingredient name as input. Generally, data from DrugBank and CTD databases was highly reliable, because it always has been well documented by experiments. The putative targets were extracted from two frequently used database platforms, STITCH (Szklarczyk et al., 2016) and Swiss Target Prediction (Gfeller et al., 2014). The reliability of the putative targets is often evaluated by the index of confidence (range from 0 to 1). The closer the confidence score is to 1, the more reliable the putative target is. However, if the threshold of the confidence is set too high, it is difficult to obtain sufficient data. Therefore, here, to make a better compromise between the quality and quantity of the data, we set the threshold values of data from STITCH and Swiss Target Prediction as 0.70 and 0.85, respectively. After removing the duplicates and those non-human targets, the remaining targets were further analyzed.

### 4.4 Collection of NAFLD-related targets

The NAFLD-related targets were collected from three existing resources, including DisGeNET (Piñero et al., 2017), CTD (Davis et al., 2009), and NCBI-gene (https://www.ncbi.nlm.nih.gov/gene/). We searched these databases by the keyword of “Non-alcoholic Fatty Liver Disease,” and only those human-related and non-repeated targets were taken into consideration. In addition, to improve the reliability of the data from CTD, only those targets curated by the experts were extracted.

### 4.5 Construction of the “ingredient-target” network

Firstly, consistency analysis between the NAFLD-related targets and BS-related targets was conducted based on the online analysis tool Venn Diagram (http://bioinformatics.psb.ugent.be/webtools/Venn/), by which we aimed at identifying the common targets between NAFLD and BS. Then network visualization tool Gephi (version 0.9.2) was utilized to construct the “ingredient-target” network by taking the common targets and the common targets-related compounds as input.

### 4.6 Tissue-specific expression pattern analysis

The tissue-specific pattern of mRNA expression can indicate important clues about gene function. BioGPS (Wu et al., 2016) is a centralized gene-annotation portal. One of its most important function is that enables researchers to access the mRNA expression data of genes in 84 organs. Herein, to investigate the distribution of the common targets attained in Section 4.5 within the liver, we ranked the target expression patterns based on their expression levels in the liver. To be specific, for certain target, if it expressed highest in the liver among the 84 organs, it was ranked 1. Inversely, if it expressed lowest in the liver, it was ranked 84.

### 4.7 Subcellular localization analysis

As we all known that protein activities are tightly linked to the cellular compartment and microenvironment where they are present. Elucidating the subcellular localization of proteins contributes to deeply understand their biological functions. Here, The Human Protein Atlas (Pontén et al., 2008), a large-scale biological database aiming at mapping the entire human proteome, was utilized to perform the subcellular localization analysis.

### 4.8 Construction of the protein-protein interaction (PPI) network

Protein-protein association network is of great value to understand the biological phenomena. As a free and publicly available source, STRING (Szklarczyk et al., 2023) database providing a comprehensive and objective global network where both the direct (physical) and indirect (functional) interactions between proteins were included. In the current study, the protein-protein interaction (PPI) information was extracted from this global network by using the common targets between NAFLD and BS as input. The PPI information with confidence score <0.4 was removed, and the remaining data was input into the network visualization tool Gephi (version 0.9.2) to obtain the PPI network map.

### 4.9 Identification of the hub genes

The importance of nodes in a network was often inferred based on their network topological features. As a powerful Cytoscape plugin, CytoHubba (Chin et al., 2014) provides at least 11 algorithms to rank nodes in a network, among which the Degree algorithm is most commonly used. Similar to the Degree algorithm, the Maximal Clique Centrality (MCC) algorithm was also proposed based on the local network topological features of nodes. Chin et al. have attempted to identify the essential proteins from the yeast PPI network by implementing multiple algorithms. As a consequence, the MCC algorithm was found to exhibit a better performance on the precision in comparison with other algorithms (Chin et al., 2014). To integrate the advantages of different algorithms, both Degree and MCC algorithms were adopted. Besides, Edge Percolated Component (EPC) algorithm was also implemented. Different from the Degree and MCC algorithms, EPC algorithm was a global network topological features-based algorithm. For each algorithm, the top 10 genes were collected, and the common genes of those three algorithms mentioned above were defined as hub genes.

### 4.10 Microarray data analysis

Firstly, hepatic gene expression data (GSE89632) in patients with NAFLD and in healthy donors were downloaded from Gene Expression Omnibus (GEO) database (https://www.ncbi.nlm.nih.gov/geo/). Chip GSE89632 was from platform GPL14951 and contained 24 healthy controls (HC), 20 simple steatosis samples (SS), and 19 nonalcoholic steatohepatitis (NASH) samples. Then GEO2R analysis (https://www.ncbi.nlm.nih.gov/geo/geo2r/) was performed to identify genes that are differentially expressed between the NAFLD patients and the healthy controls. The detailed screening criteria were set as *p* < 0.05 and |log(FC)| > 1. Finally, we compared the differentially expressed genes with the hub genes obtained in Section 4.9, and the common genes between these two datasets were further analyzed.

### 4.11 Molecular docking analysis

To investigate the interactions between the active compounds and the key targets, molecular docking analysis was performed based on AutoDock Vina (version 1.1.2). Here, the 2D structures of the active compounds were obtained from PubChem database, and the X-ray crystallography-based structures of the key targets were downloaded from RCSB Protein Data Bank. When we implemented the AutoDock program, the size of the Grid Box was set to 40 × 40 × 40, and the exhaustiveness value was set as 100. Finally, the docking complexes with the lowest binding energy were selected for further analysis.

### 4.12 Molecular dynamics (MD) simulations

MD simulation is an efficient method to evaluate the stability of the protein-ligand complexes. In present study, MD simulation was carried out based on AMBER 18 software package with the force fields of FF14SB and GAFF. For each studied docked complex, a 100 ns MD simulation was performed under the condition of constant temperature. Finally, several important indices, including Root Mean Square Deviation (RMSD), Mean Square Fluctuation (RMSF) and binding free energy were calculated, by which we attempted to evaluate the stability of the docked complexes comprehensively. Of note, here, the binding free energy was calculated based on the last 50ns trajectories by utilizing the MMPBSA. py module.

### 4.13 Modular analysis of the PPI network

Molecular network is a biological system which consisted of a large number of nodes and edges. The characteristic of high complexity making it to be a challenging work to elucidate its scientific connotation. Network module theory holds the view that biological system network has modularity, and the nodes in the same module always perform some biological functions cooperatively (Lorenz et al., 2011). Therefore, modular analysis is considered to be an effective method to analyze the complex biological network. During the past decade, dozens of modular analysis methods have been proposed. To solve the problem with optimization of the network module division, Gu et al. proposed a network structure entropy-based method to evaluate the effect of 11 commonly used module division methods. As a result, Molecular Complex Detection (MCODE) algorithm was found to be superior to other algorithms (Gu et al., 2018). Therefore, in present study, the MCODE algorithm was implemented to identify the functional modules of BS on NAFLD.

### 4.14 Kyoto Encyclopedia of genes and genomes (KEGG) pathway enrichment analysis

To identify the pathways involved in BS on NAFLD, the platform of Database for Annotation, Visualization and Integrated Discovery (DAVID) (Dennis et al., 2003) was utilized to perform Kyoto Encyclopedia of Genes and Genomes (KEGG) enrichment analysis, and only those pathways of which adjusted *p*-values <0.05 were taken into consideration. In addition, for each pathway attained, we annotated its biological functions systematically by summarizing the related information recorded in literature and databases.

All of the databases utilized in the current study were listed in Table 5 in detail, which would assist the reader to understand our research methods more easily and quickly.

**TABLE 5:** A summary of the databases and analysis tools utilized in the current study.

ID	Data/analysis type	Source/tool
1	Chemical component of BS	TCMSP, TCMID, ETCM
2	DL and OB of chemical component	TCMSP, ETCM
3	BS-related targets	DrugBank, CTD, STITCH, Swiss Target Prediction
4	NAFLD-related targets	DisGeNET, CTD, NCBI-gene
5	Tissue-specific expression pattern analysis	BioGPS
6	Subcellular localization analysis	The Human Protein Atlas
7	Protein-protein interaction	STRING
8	Identification of the hub genes	CytoHubba
9	Microarray data analysis	Gene Expression Omnibus, GEO2R
10	Molecular docking analysis	AutoDock Vina (version 1.1.2)
11	Molecular Dynamics Simulations	AMBER 18 software package
12	Modular analysis	Cytoscape, MCODE algorithm
13	KEGG pathway enrichment analysis	DAVID
14	Network visualization	Gephi (version 0.9.2)

## 5 Conclusion

In present study, to clarify the material basis and mechanism of BS against NAFLD in a precise manner, an original computational pharmacology method for identifying the hepatoprotective ingredient group of TCMs was proposed. Then by incorporating with the techniques of network pharmacology, molecular docking, and molecular dynamics simulation, the proposed computational pharmacology method was utilized to reveal the scientific connotation of BS on NAFLD from multiple perspectives, including active ingredients, key targets, key pathways and the major biological processes involved. As a result, a total of 12 ingredients, mainly including monoterpene glucosides, flavonoids, triterpenes, and phenolic acids, were found to be associated with the anti-NAFLD effect of BS. Hub gene analysis and microarray data analysis indicated that IL1-β, IL6, and JUN were the key targets of BS on NAFLD. The findings mentioned above were then further validated via molecular docking analysis, molecular dynamics simulation analysis, and literature analysis. In addition, the key KEGG pathways and the major biological processes of BS on NAFLD were also identified. It’s worth noting that the NAFLD pathway was significantly enriched. Further analysis found that there was a great deal of evidence available in the literature to support the regulatory effect of BS on NAFLD pathway. In addition, inflammation, apoptosis, lipid metabolism, and glucose metabolism were found to play critical roles in the process of BS alleviate NAFLD. In summary, a novel and effective bioinformatics strategy was proposed to uncover the material basis and mechanism of TCM in this work, based on which the anti-NAFLD effect of BS was systematically investigated from multiple perspectives.

## Data Availability

The original contributions presented in the study are included in the article/Supplementary Material, further inquiries can be directed to the corresponding authors.

## References

[B1] Akiyama M. Mizokami T. Ito H. Ikeda Y. (2023). A randomized, placebo-controlled trial evaluating the safety of excessive administration of kaempferol aglycone. Food Sci. Nutr. 11 (9), 5427–5437. 10.1002/fsn3.3499 37701215 PMC10494647

[B2] Breikaa R. M. Algandaby M. M. El-Demerdash E. Abdel-Naim A. B. (2013a). Biochanin A protects against acute carbon tetrachloride-induced hepatotoxicity in rats. Biosci. Biotechnol. Biochem. 77 (5), 909–916. 10.1271/bbb.120675 23649249

[B3] Breikaa R. M. Algandaby M. M. El-Demerdash E. Abdel-Naim A. B. (2013b). Multimechanistic antifibrotic effect of biochanin a in rats: implications of proinflammatory and profibrogenic mediators. PLoS One 8 (7), e69276. 10.1371/journal.pone.0069276 23874933 PMC3712926

[B4] Canbay A. Gieseler R. K. Gores G. J. Gerken G. (2005). The relationship between apoptosis and non-alcoholic fatty liver disease: an evolutionary cornerstone turned pathogenic. Z Gastroenterol. 43 (2), 211–217. 10.1055/s-2004-813744 15700216

[B5] Cao Z. X. Chen Y. L. Li W. X. Wang X. Y. Niu L. Jia W. H. (2022). Study on the toxicological mechanism of Psoraleae Fructus induced liver injury based on network pharmacology and cell experiment. Chin. J. Mod. Appl. Pharm. 39 (16), 2052–2062. 10.13748/j.cnki.issn1007-7693.2022.16.002

[B6] Chalasani N. Younossi Z. Lavine J. E. Diehl A. M. Brunt E. M. Cusi K. (2012). The diagnosis and management of non-alcoholic fatty liver disease: practice guideline by the American association for the study of liver diseases, American college of gastroenterology, and the American gastroenterological association. Hepatology 55 (6), 2005–2023. 10.1002/hep.25762 22488764

[B7] Chang K. F. Huang X. F. Chang J. T. Huang Y. C. Lo W. S. Hsiao C. Y. (2020). Cedrol, a sesquiterpene alcohol, enhances the anticancer efficacy of temozolomide in attenuating drug resistance via regulation of the DNA damage response and MGMT expression. J. Nat. Prod. 83 (10), 3021–3029. 10.1021/acs.jnatprod.0c00580 32960603

[B8] Chao H. W. Chao S. W. Lin H. Ku H. C. Cheng C. F. (2019). Homeostasis of glucose and lipid in non-alcoholic fatty liver disease. Int. J. Mol. Sci. 20 (2), 298. 10.3390/ijms20020298 30642126 PMC6359196

[B9] Chen J. M. Zhang S. S. Guo Q. K. Li L. Wu Z. Y. Wang Z. F. (2013). Study on the curative effect and the protection mechanism of Paeonifl orin on non-alcoholic fatty liver rats. China J. Traditional Chin. Med. Pharm. 28 (05), 1376–1381.

[B10] Chen L. Wei S. Liu H. Li J. Jing M. Tong Y. (2021). Paeoniflorin protects against ANIT-induced cholestatic liver injury in rats via the activation of SIRT1-FXR signaling pathway. Evid. Based Complement. Altern. Med. 2021, 8479868. 10.1155/2021/8479868 PMC8429014 34512782

[B11] Chen M. Cao L. Luo Y. Feng X. Sun L. Wen M. (2015a). Paeoniflorin protects against concanavalin A-induced hepatitis in mice. Int. Immunopharmacol. 24 (1), 42–49. 10.1016/j.intimp.2014.11.006 25479726

[B12] Chen Z. Ma X. Zhu Y. Zhao Y. Wang J. Li R. (2015b). Paeoniflorin ameliorates ANIT-induced cholestasis by activating Nrf2 through an PI3K/Akt-dependent pathway in rats. Phytother. Res. 29 (11), 1768–1775. 10.1002/ptr.5431 26269092

[B13] Chin C. H. Chen S. H. Wu H. H. Ho C. W. Ko M. T. Lin C. Y. (2014). cytoHubba: identifying hub objects and sub-networks from complex interactome. BMC Syst. Biol. 8 (Suppl. 4), S11. 10.1186/1752-0509-8-s4-s11 25521941 PMC4290687

[B14] Chinese Pharmacopeia Commission (2020). Pharmacopoeia of people's Republic of China. Beijing: China Medical Science and Technology Press.

[B15] Davis A. P. Murphy C. G. Saraceni-Richards C. A. Rosenstein M. C. Wiegers T. C. Mattingly C. J. (2009). Comparative Toxicogenomics Database: a knowledgebase and discovery tool for chemical-gene-disease networks. Nucleic Acids Res. 37 (Database issue), D786–D792. 10.1093/nar/gkn580 18782832 PMC2686584

[B16] Deng X. Li Y. Li X. Zhang Z. Dai S. Wu H. (2022). Paeoniflorin protects against acetaminophen-induced liver injury in mice via JNK signaling pathway. Molecules 27 (23), 8534. 10.3390/molecules27238534 36500627 PMC9739375

[B17] DennisG.Jr.ShermanB. T.HosackD. A.YangJ.GaoW.LaneH. C. (2003). DAVID: database for annotation, visualization, and integrated discovery. Genome Biol. 4 (5), R60. 10.1186/gb-2003-4-9-r60 12734009

[B18] Domitrović R. Jakovac H. (2010). Antifibrotic activity of anthocyanidin delphinidin in carbon tetrachloride-induced hepatotoxicity in mice. Toxicology 272 (1-3), 1–10. 10.1016/j.tox.2010.03.016 20371262

[B19] Estes C. Razavi H. Loomba R. Younossi Z. Sanyal A. J. (2018). Modeling the epidemic of nonalcoholic fatty liver disease demonstrates an exponential increase in burden of disease. Hepatology 67 (1), 123–133. 10.1002/hep.29466 28802062 PMC5767767

[B20] Ezhilarasan D. Shree Harini K. Karthick M. Selvaraj C. (2024). Ethyl gallate concurrent administration protects against acetaminophen-induced acute liver injury in mice: an *in vivo* and *in silico* approach. Chem. Biol. Drug Des. 103 (1), e14369. 10.1111/cbdd.14369 37817304

[B21] Ezquerro S. Mocha F. Frühbeck G. Guzmán-Ruiz R. Valentí V. Mugueta C. (2019). Ghrelin reduces TNF-α-induced human hepatocyte apoptosis, autophagy, and pyroptosis: role in obesity-associated NAFLD. J. Clin. Endocrinol. Metab. 104 (1), 21–37. 10.1210/jc.2018-01171 30137403

[B22] Fan Y. Yan L. T. Yao Z. Xiong G. Y. (2021). Biochanin A regulates cholesterol metabolism further delays the progression of nonalcoholic fatty liver disease. Diabetes Metab. Syndr. Obes. 14, 3161–3172. 10.2147/dmso.s315471 34276221 PMC8277457

[B23] Gao M. Zhang D. Jiang C. Jin Q. Zhang J. (2023). Paeoniflorin inhibits hepatocellular carcinoma growth by reducing PD-L1 expression. Biomed. Pharmacother. 166, 115317. 10.1016/j.biopha.2023.115317 37597322

[B24] Gfeller D. Grosdidier A. Wirth M. Daina A. Michielin O. Zoete V. (2014). SwissTargetPrediction: a web server for target prediction of bioactive small molecules. Nucleic Acids Res. 42 (Web Server issue), W32–W38. 10.1093/nar/gku293 24792161 PMC4086140

[B25] Ghosh N. Ghosh R. Mandal V. Mandal S. C. (2011). Recent advances in herbal medicine for treatment of liver diseases. Pharm. Biol. 49 (9), 970–988. 10.3109/13880209.2011.558515 21595500

[B26] Girish C. Pradhan S. C. (2012). Indian herbal medicines in the treatment of liver diseases: problems and promises. Fundam. Clin. Pharmacol. 26 (2), 180–189. 10.1111/j.1472-8206.2011.01011.x 22136107

[B27] Gu H. Chen Y. Y. Wang P. Q. Wang Z. (2018). Comparison of different methods of module division by entropy and functional similarity of gene network and its modules for coronary heart disease. Chin. J. Pharmacol. Toxicol. 32 (05), 377–384.

[B28] Guo C. Zheng L. Chen S. Liang X. Song X. Wang Y. (2023). Thymol ameliorates ethanol-induced hepatotoxicity via regulating metabolism and autophagy. Chem. Biol. Interact. 370, 110308. 10.1016/j.cbi.2022.110308 36535314

[B29] He P. Yu Z. J. Sun C. Y. Jiao S. J. Jiang H. Q. (2016). Knockdown of eIF3a attenuates the pro-fibrogenic response of hepatic stellate cells induced by TGF-β1. Cell. Mol. Biol. (Noisy-le-grand) 62 (6), 107–111. 27262813

[B30] He S. Yi Y. Hou D. Fu X. Zhang J. Ru X. (2022). Identification of hepatoprotective traditional Chinese medicines based on the structure-activity relationship, molecular network, and machine learning techniques. Front. Pharmacol. 13, 969979. 10.3389/fphar.2022.969979 36105213 PMC9465166

[B31] Hong M. Li S. Tan H. Y. Cheung F. Wang N. Huang J. (2017). A network-based pharmacology study of the herb-induced liver injury potential of traditional hepatoprotective Chinese herbal medicines. Molecules 22 (4), 632. 10.3390/molecules22040632 28420096 PMC6154655

[B32] Hopkins A. L. (2008). Network pharmacology: the next paradigm in drug discovery. Nat. Chem. Biol. 4 (11), 682–690. 10.1038/nchembio.118 18936753

[B33] Hu B. An H. M. Wang S. S. Chen J. J. Xu L. (2016). Preventive and therapeutic effects of Chinese herbal compounds against hepatocellular carcinoma. Molecules 21 (2), 142. 10.3390/molecules21020142 26828466 PMC6274246

[B34] Jafari A. Rasmi Y. Hajaghazadeh M. Karimipour M. (2018). Hepatoprotective effect of thymol against subchronic toxicity of titanium dioxide nanoparticles: biochemical and histological evidences. Environ. Toxicol. Pharmacol. 58, 29–36. 10.1016/j.etap.2017.12.010 29289817

[B35] Jeong M. Y. Park J. Youn D. H. Jung Y. Kang J. Lim S. (2017). Albiflorin ameliorates obesity by inducing thermogenic genes via AMPK and PI3K/AKT *in vivo* and *in vitro* . Metabolism 73, 85–99. 10.1016/j.metabol.2017.05.009 28732574

[B36] Ji J. (2022). Study on the mechanism of total glycosides of peony against non-alcoholic fatty liver via regulating lipid metabolism and inflammaion. Beijing: Beijing University of Chinese Medicine.

[B37] Jiang H. Li J. Wang L. Wang S. Nie X. Chen Y. (2020). Total glucosides of paeony: a review of its phytochemistry, role in autoimmune diseases, and mechanisms of action. J. Ethnopharmacol. 258, 112913. 10.1016/j.jep.2020.112913 32371143

[B38] Kaur P. Mehta R. G. Arora S. Singh B. (2018). Progression of conventional hepatic cell culture models to bioengineered HepG2 cells for evaluation of herbal bioactivities. Biotechnol. Lett. 40 (6), 881–893. 10.1007/s10529-018-2547-y 29616383

[B39] Kibble M. Saarinen N. Tang J. Wennerberg K. Makela S. Aittokallio T. (2015). Network pharmacology applications to map the unexplored target space and therapeutic potential of natural products. Nat. Prod. Rep. 32 (8), 1249–1266. 10.1039/c5np00005j 26030402

[B40] Kim M. H. Park J. S. Jung J. W. Byun K. W. Kang K. S. Lee Y. S. (2011). Daidzein supplementation prevents non-alcoholic fatty liver disease through alternation of hepatic gene expression profiles and adipocyte metabolism. Int. J. Obes. (Lond). 35 (8), 1019–1030. 10.1038/ijo.2010.256 21157426

[B41] Laddha A. P. Murugesan S. Kulkarni Y. A. (2022). *In-vivo* and in-silico toxicity studies of daidzein: an isoflavone from soy. Drug Chem. Toxicol. 45 (3), 1408–1416. 10.1080/01480545.2020.1833906 33059469

[B42] Lahmi A. Oryan S. Eidi A. Rohani A. H. (2023). Comparative effects of thymol and vitamin E on nonalcoholic fatty liver disease in male Wistar rats. Braz J. Biol. 84, e268781. 10.1590/1519-6984.268781 36629640

[B43] Li L. Wang H. Zhao S. Zhao Y. Chen Y. Zhang J. (2022). Paeoniflorin ameliorates lipopolysaccharide-induced acute liver injury by inhibiting oxidative stress and inflammation via SIRT1/FOXO1a/SOD2 signaling in rats. Phytother. Res. 36 (6), 2558–2571. 10.1002/ptr.7471 35570830

[B44] Li S. (2016). Exploring traditional Chinese medicine by a novel therapeutic concept of network target. Chin. J. Integr. Med. 22 (9), 647–652. 10.1007/s11655-016-2499-9 27145941

[B45] Li S. Wang Y. Li C. Yang N. Yu H. Zhou W. (2021). Study on hepatotoxicity of rhubarb based on metabolomics and network pharmacology. Drug Des. Devel Ther. 15, 1883–1902. 10.2147/dddt.s301417 PMC8106470 33976539

[B46] Li S. Zhang B. (2013). Traditional Chinese medicine network pharmacology: theory, methodology and application. Chin. J. Nat. Med. 11 (2), 110–120. 10.1016/S1875-5364(13)60037-0 23787177

[B47] Li Y. Hai J. Li L. Chen X. Peng H. Cao M. (2013). Administration of ghrelin improves inflammation, oxidative stress, and apoptosis during and after non-alcoholic fatty liver disease development. Endocrine 43 (2), 376–386. 10.1007/s12020-012-9761-5 22843123

[B48] Li Y. C. Qiao J. Y. Wang B. Y. Bai M. Shen J. D. Cheng Y. X. (2018). Paeoniflorin ameliorates fructose-induced insulin resistance and hepatic steatosis by activating LKB1/AMPK and AKT pathways. Nutrients 10 (8), 1024. 10.3390/nu10081024 30081580 PMC6116094

[B49] Li Y. M. Bao Y. Y. He H. W. Zhang N. (2023). Activity and mechanism of palbinone against hepatic fibrosis and inflammation. Acta Pharm. Sin. 58 (02), 371–376. 10.16438/j.0513-4870.2022-1050

[B50] Liu T. Zhang N. Kong L. Chu S. Zhang T. Yan G. (2022). Paeoniflorin alleviates liver injury in hypercholesterolemic rats through the ROCK/AMPK pathway. Front. Pharmacol. 13, 968717. 10.3389/fphar.2022.968717 36081948 PMC9445162

[B51] Liu Y. He C. Y. Yang X. M. Chen W. C. Zhang M. J. Zhong X. D. (2023). Paeoniflorin coordinates macrophage polarization and mitigates liver inflammation and fibrogenesis by targeting the NF-[Formula: see text]B/HIF-1α pathway in CCl_4_-induced liver fibrosis. Am. J. Chin. Med. 51 (5), 1249–1267. 10.1142/s0192415x2350057x 37317554

[B52] Lorenz D. M. Jeng A. Deem M. W. (2011). The emergence of modularity in biological systems. Phys. Life Rev. 8 (2), 129–160. 10.1016/j.plrev.2011.02.003 21353651 PMC4477837

[B53] Lu X. Song M. Gao N. (2023). Extracellular vesicles and fatty liver. Adv. Exp. Med. Biol. 1418, 129–141. 10.1007/978-981-99-1443-2_9 37603277

[B54] Ma X. Wen J. X. Gao S. J. He X. Li P. Y. Yang Y. X. (2018). Paeonia lactiflora Pall. regulates the NF-κB-NLRP3 inflammasome pathway to alleviate cholestasis in rats. J. Pharm. Pharmacol. 70 (12), 1675–1687. 10.1111/jphp.13008 30277564

[B55] Ma X. Zhang W. Jiang Y. Wen J. Wei S. Zhao Y. (2020). Paeoniflorin, a natural product with multiple targets in liver diseases-A mini review. Front. Pharmacol. 11, 531. 10.3389/fphar.2020.00531 32410996 PMC7198866

[B56] Ma X. Zhao Y. Yang T. Gong N. Chen X. Liu G. (2022). Integration of network pharmacology and molecular docking to explore the molecular mechanism of Cordycepin in the treatment of Alzheimer's disease. Front. Aging Neurosci. 14, 1058780. 10.3389/fnagi.2022.1058780 36620771 PMC9817107

[B57] Ma X. Zhao Y. L. Zhu Y. Chen Z. Wang J. B. Li R. Y. (2015). Paeonia lactiflora Pall. protects against ANIT-induced cholestasis by activating Nrf2 via PI3K/Akt signaling pathway. Drug Des. Devel Ther. 9, 5061–5074. 10.2147/dddt.s90030 PMC4562737 26366057

[B58] Ma Z. Chu L. Liu H. Li J. Zhang Y. Liu W. (2016). Paeoniflorin alleviates non-alcoholic steatohepatitis in rats: involvement with the ROCK/NF-κB pathway. Int. Immunopharmacol. 38, 377–384. 10.1016/j.intimp.2016.06.023 27351828

[B59] Ma Z. Chu L. Liu H. Wang W. Li J. Yao W. (2017). Beneficial effects of paeoniflorin on non-alcoholic fatty liver disease induced by high-fat diet in rats. Sci. Rep. 7, 44819. 10.1038/srep44819 28300221 PMC5353673

[B60] Mantovani A. Petracca G. Beatrice G. Csermely A. Lonardo A. Schattenberg J. M. (2022). Non-alcoholic fatty liver disease and risk of incident chronic kidney disease: an updated meta-analysis. Gut 71 (1), 156–162. 10.1136/gutjnl-2020-323082 33303564

[B61] Mantovani A. Scorletti E. Mosca A. Alisi A. Byrne C. D. Targher G. (2020). Complications, morbidity and mortality of nonalcoholic fatty liver disease. Metabolism 111S, 154170. 10.1016/j.metabol.2020.154170 32006558

[B62] Musial C. Kuban-Jankowska A. Gorska-Ponikowska M. (2020). Beneficial properties of green tea catechins. Int. J. Mol. Sci. 21 (5), 1744. 10.3390/ijms21051744 32143309 PMC7084675

[B63] Oh H. Lee H. S. Kim T. Chai K. Y. Chung H. T. Kwon T. O. (2002). Furocoumarins from Angelica dahurica with hepatoprotective activity on tacrine-induced cytotoxicity in Hep G2 cells. Planta Med. 68 (5), 463–464. 10.1055/s-2002-32075 12058329

[B64] Park S. H. Hong J. Y. Park H. J. Lee S. K. (2020). The antiproliferative activity of oxypeucedanin via induction of G(2)/M phase cell cycle arrest and p53-dependent MDM2/p21 expression in human hepatoma cells. Molecules 25 (3), 501. 10.3390/molecules25030501 31979361 PMC7037124

[B65] Patel R. Mueller M. (2024). Alcoholic liver disease. Treasure Island (FL): StatPearls Publishing. 31536239

[B66] Peng L. Ma Z. Chu W. Jiang P. Fu Y. Wang P. (2023). Identification and hepatoprotective activity of total glycosides of paeony with high content of paeoniflorin extracted from Paeonia lactiflora Pall. Food Chem. Toxicol. 173, 113624. 10.1016/j.fct.2023.113624 36681265

[B67] Perumpail B. J. Khan M. A. Yoo E. R. Cholankeril G. Kim D. Ahmed A. (2017). Clinical epidemiology and disease burden of nonalcoholic fatty liver disease. World J. Gastroenterol. 23 (47), 8263–8276. 10.3748/wjg.v23.i47.8263 29307986 PMC5743497

[B68] Piñero J. Bravo À. Queralt-Rosinach N. Gutiérrez-Sacristán A. Deu-Pons J. Centeno E. (2017). DisGeNET: a comprehensive platform integrating information on human disease-associated genes and variants. Nucleic Acids Res. 45 (D1), D833-D839–D839. 10.1093/nar/gkw943 27924018 PMC5210640

[B69] Pontén F. Jirström K. Uhlen M. (2008). The human protein atlas--a tool for pathology. J. Pathol. 216 (4), 387–393. 10.1002/path.2440 18853439

[B70] Powell E. E. Wong V. W. Rinella M. (2021). Non-alcoholic fatty liver disease. Lancet 397 (10290), 2212–2224. 10.1016/s0140-6736(20)32511-3 33894145

[B71] Qin Y. Tian Y. P. (2011). Protective effects of total glucosides of paeony and the underlying mechanisms in carbon tetrachloride-induced experimental liver injury. Arch. Med. Sci. 7 (4), 604–612. 10.5114/aoms.2011.24129 22291795 PMC3258771

[B72] Qiu Y. Liu S. Chen H. T. Yu C. H. Teng X. D. Yao H. T. (2013). Upregulation of caveolin-1 and SR-B1 in mice with non-alcoholic fatty liver disease. Hepatobiliary Pancreat. Dis. Int. 12 (6), 630–636. 10.1016/s1499-3872(13)60099-5 24322749

[B73] Rao S. S. Hu Y. Xie P. L. Cao J. Wang Z. X. Liu J. H. (2018). Omentin-1 prevents inflammation-induced osteoporosis by downregulating the pro-inflammatory cytokines. Bone Res. 6, 9. 10.1038/s41413-018-0012-0 29619269 PMC5876344

[B74] Ren J. Lu Y. Qian Y. Chen B. Wu T. Ji G. (2019). Recent progress regarding kaempferol for the treatment of various diseases. Exp. Ther. Med. 18 (4), 2759–2776. 10.3892/etm.2019.7886 31572524 PMC6755486

[B75] Rinella M. E. Sanyal A. J. (2016). Management of NAFLD: a stage-based approach. Nat. Rev. Gastroenterol. Hepatol. 13 (4), 196–205. 10.1038/nrgastro.2016.3 26907882

[B76] Ru J. Li P. Wang J. Zhou W. Li B. Huang C. (2014). TCMSP: a database of systems pharmacology for drug discovery from herbal medicines. J. Cheminform 6, 13. 10.1186/1758-2946-6-13 24735618 PMC4001360

[B77] Sherman D. J. Liu L. Mamrosh J. L. Xie J. Ferbas J. Lomenick B. (2023). The fatty liver disease-causing protein PNPLA3-I148M alters lipid droplet-Golgi dynamics. bioRxiv, 562302. 10.1101/2023.10.13.562302 PMC11067037 38657050

[B78] Shin M. R. Lee S. H. Roh S. S. (2022). The potential hepatoprotective effect of Paeoniae Radix Alba in thioacetamide-induced acute liver injury in rats. Evid. Based Complement. Altern. Med. 2022, 7904845. 10.1155/2022/7904845 PMC8816603 35126604

[B79] Siegel R. Naishadham D. Jemal A. (2012). Cancer statistics, 2012. CA Cancer J. Clin. 62 (1), 10–29. 10.3322/caac.20138 22237781

[B80] Sun S. Xu K. Yan M. Cui J. Zhu K. Yang Y. (2023). Delphinidin induces autophagic flux blockage and apoptosis by inhibiting both multidrug resistance gene 1 and DEAD-box helicase 17 expressions in liver cancer cells. J. Pharm. Pharmacol. 75 (2), 253–263. 10.1093/jpp/rgac037 36179123

[B81] Sun Z. J. Ye Y. M. Wang G. X. (2022). Study on the action mechanism of total glycosides of peony against non-alcoholic fatty liver *in vitro* . Mod. Traditional Chin. Med. Materia Medica-World Sci. Technol. 24 (07), 2748–2754. 10.11842/wst.20210917003

[B82] Szklarczyk D. Kirsch R. Koutrouli M. Nastou K. Mehryary F. Hachilif R. (2023). The STRING database in 2023: protein-protein association networks and functional enrichment analyses for any sequenced genome of interest. Nucleic Acids Res. 51 (D1), D638–D646. 10.1093/nar/gkac1000 36370105 PMC9825434

[B83] Szklarczyk D. Santos A. von Mering C. Jensen L. J. Bork P. Kuhn M. (2016). STITCH 5: augmenting protein-chemical interaction networks with tissue and affinity data. Nucleic Acids Res. 44 (D1), D380–D384. 10.1093/nar/gkv1277 26590256 PMC4702904

[B84] Tian T. L. Zhu M. H. Cheng D. W. Li T. J. (2014). Clinical study on the combination of polyene phosphatidylcholine and total glycosides of peony in the treatment of alcoholic fatty liver disease. Jiangsu J. Traditional Chin. Med. 46 (12), 24–26.

[B85] Van Puyvelde L. Kayonga A. Brioen P. Costa J. Ndimubakunzi A. De Kimpe N. (1989). The hepatoprotective principle of Hypoestes triflora leaves. J. Ethnopharmacol. 26 (2), 121–127. 10.1016/0378-8741(89)90059-7 2601353

[B86] Vinholes J. Rudnitskaya A. Gonçalves P. Martel F. Coimbra M. A. Rocha S. M. (2014). Hepatoprotection of sesquiterpenoids: a quantitative structure-activity relationship (QSAR) approach. Food Chem. 146, 78–84. 10.1016/j.foodchem.2013.09.039 24176316

[B87] Wang F. Liu Y. Dong Y. Zhao M. Huang H. Jin J. (2023a). Haploinsufficiency of Lipin3 leads to hypertriglyceridemia and obesity by disrupting the expression and nucleocytoplasmic localization of Lipin1. Front. Med. 18, 180–191. 10.1007/s11684-023-1003-0 37776435

[B88] Wang H. (2005). Anti-hapatofibrotic effects of total glucosides of paeony on hepatic fibrosis and G protein-coupled signal transduction of hepatic stellate cell. Anhui: Anhui Medical University.

[B89] Wang Y. Cheng C. Zhao T. Cao J. Liu Y. Wang Y. (2023b). Phytochemicals from anneslea fragrans wall. And their hepatoprotective and anti-inflammatory activities. Molecules 28 (14), 5480. 10.3390/molecules28145480 37513352 PMC10384535

[B90] Wang Y. Y. Li J. Wu Z. R. Zhang B. Yang H. B. Wang Q. (2017). Insights into the molecular mechanisms of Polygonum multiflorum Thunb-induced liver injury: a computational systems toxicology approach. Acta Pharmacol. Sin. 38 (5), 719–732. 10.1038/aps.2016.147 28239160 PMC5457689

[B91] Wishart D. S. Knox C. Guo A. C. Cheng D. Shrivastava S. Tzur D. (2008). DrugBank: a knowledgebase for drugs, drug actions and drug targets. Nucleic Acids Res. 36 (Database issue), D901–D906. 10.1093/nar/gkm958 18048412 PMC2238889

[B92] Wu C. Jin X. Tsueng G. Afrasiabi C. Su A. I. (2016). BioGPS: building your own mash-up of gene annotations and expression profiles. Nucleic Acids Res. 44 (D1), D313–D316. 10.1093/nar/gkv1104 26578587 PMC4702805

[B93] Xiao X. Hu Q. Deng X. Shi K. Zhang W. Jiang Y. (2022). Old wine in new bottles: kaempferol is a promising agent for treating the trilogy of liver diseases. Pharmacol. Res. 175, 106005. 10.1016/j.phrs.2021.106005 34843960

[B94] Xiao Y. Gong Q. Wang W. Liu F. Kong Q. Pan F. (2020). The combination of Biochanin A and SB590885 potentiates the inhibition of tumour progression in hepatocellular carcinoma. Cancer Cell. Int. 20, 371. 10.1186/s12935-020-01463-w 32774165 PMC7405455

[B95] Xu H. Y. Zhang Y. Q. Liu Z. M. Chen T. Lv C. Y. Tang S. H. (2019). ETCM: an encyclopaedia of traditional Chinese medicine. Nucleic Acids Res. 47 (D1), D976-D982–D982. 10.1093/nar/gky987 30365030 PMC6323948

[B96] Xue R. Fang Z. Zhang M. Yi Z. Wen C. Shi T. (2013). TCMID: traditional Chinese Medicine integrative database for herb molecular mechanism analysis. Nucleic Acids Res. 41 (Database issue), D1089–D1095. 10.1093/nar/gks1100 23203875 PMC3531123

[B97] Yang Y. L. Pan J. Q. Tan S. Z. Zheng L. Y. Huang Z. Cong L. L. (2017). Effect of total glucosides of paeony on omentin and TLR4 in liver tissue of NAFLD rat. Pharmacol. Clin. Chin. Materia Medica 33 (03), 62–65. 10.13412/j.cnki.zyyl.2017.03.019

[B98] Yao H. Qiao Y. J. Zhao Y. L. Tao X. F. Xu L. N. Yin L. H. (2016). Herbal medicines and nonalcoholic fatty liver disease. World J. Gastroenterol. 22 (30), 6890–6905. 10.3748/wjg.v22.i30.6890 27570425 PMC4974587

[B99] Yoshitaka H. Hamaguchi M. Kojima T. Fukuda T. Ohbora A. Fukui M. (2017). Nonoverweight nonalcoholic fatty liver disease and incident cardiovascular disease: a *post hoc* analysis of a cohort study. Med. Baltim. 96 (18), e6712. 10.1097/md.0000000000006712 PMC5419911 28471965

[B100] Younossi Z. M. Koenig A. B. Abdelatif D. Fazel Y. Henry L. Wymer M. (2016). Global epidemiology of nonalcoholic fatty liver disease-Meta-analytic assessment of prevalence, incidence, and outcomes. Hepatology 64 (1), 73–84. 10.1002/hep.28431 26707365

[B101] Yu Z. Yang L. Deng S. Liang M. (2020). Daidzein ameliorates LPS-induced hepatocyte injury by inhibiting inflammation and oxidative stress. Eur. J. Pharmacol. 885, 173399. 10.1016/j.ejphar.2020.173399 32712091

[B102] Zakaria S. Nawaya R. Abdel-Hamid N. M. Eldomany R. A. El-Shishtawy M. M. (2021). Targeting the HIF-1α/Cav-1 pathway with a chicory extract/daidzein combination plays a potential role in retarding hepatocellular carcinoma. Curr. Cancer Drug Targets 21 (10), 881–896. 10.2174/1568009621666210811121120 34382525

[B103] Zhang J. Fu Y. Yang B. Xiang X. (2022a). Total glucosides of paeony inhibits liver fibrosis and inflammatory response associated with cirrhosis via the FLI1/NLRP3 axis. Am. J. Transl. Res. 14 (6), 4321–4336. 35836848 PMC9274563

[B104] Zhang L. Yang B. Yu B. (2015). Paeoniflorin protects against nonalcoholic fatty liver disease induced by a high-fat diet in mice. Biol. Pharm. Bull. 38 (7), 1005–1011. 10.1248/bpb.b14-00892 25972092

[B105] Zhang Y. Zhang S. Luo X. Zhao H. Xiang X. (2022b). Paeoniflorin mitigates PBC-induced liver fibrosis by repressing NLRP3 formation. Acta Cir. Bras. 36 (11), e361106. 10.1590/acb361106 35195182 PMC8860402

[B106] Zhao Y. He X. Ma X. Wen J. Li P. Wang J. (2017). Paeoniflorin ameliorates cholestasis via regulating hepatic transporters and suppressing inflammation in ANIT-fed rats. Biomed. Pharmacother. 89, 61–68. 10.1016/j.biopha.2017.02.025 28214689

[B107] Zhao Y. Ma X. Wang J. Zhu Y. Li R. Wang J. (2014). Paeoniflorin alleviates liver fibrosis by inhibiting HIF-1α through mTOR-dependent pathway. Fitoterapia 99, 318–327. 10.1016/j.fitote.2014.10.009 25454463

[B108] Zheng L. Q. Hui H. Tian G. Xu X. F. Qin L. P. Shan Q. Y. (2020). Study on hepatotoxicity mechanism of Euodiae Fructus based on network pharmacology. Chin. Traditional Herb. Drugs 51 (02), 419–425.

[B109] Zheng L. Y. Pan J. Q. Lv J. H. (2008). Effects of total glucosides of paeony on enhancing insulin sensitivity and antagonizing non-alcoholic fatty liver in rats. China J. Chin. Mat. Med. 33 (20), 2385–2390. 19157135

[B110] Zhou H. Q. Liu W. Wang J. Huang Y. Q. Li P. Y. Zhu Y. (2017). Paeoniflorin attenuates ANIT-induced cholestasis by inhibiting apoptosis *in vivo* via mitochondria-dependent pathway. Biomed. Pharmacother. 89, 696–704. 10.1016/j.biopha.2017.02.084 28267673

